# Development of a Tool to Detect Open-Mouthed Respiration in Caged Broilers

**DOI:** 10.3390/ani15182732

**Published:** 2025-09-18

**Authors:** Yali Ma, Yongmin Guo, Bin Gao, Pengshen Zheng, Changxi Chen

**Affiliations:** 1 Key Laboratory of Smart Breeding (Co-Construction by Ministry and Province), Ministry of Agriculture and Rural Affairs, Tianjin 300384, China; mayali_7447@163.com (Y.M.); guoyongmin8@163.com (Y.G.); bingao_1594@163.com (B.G.); 2 College of Computer and Information Engineering, Tianjin Agricultural University, Tianjin 300384, China; zhengpengshen_9206@163.com

**Keywords:** broiler open-mouth behavior, heat stress detection, small-object detection, precision livestock farming

## Abstract

Open-mouth panting in broiler chickens is a visible and critical indicator of heat stress and compromised welfare. However, detecting this behavior in densely populated cages is challenging due to the small size of the target and frequent occlusions and cluttered backgrounds. To overcome these issues, we proposed an enhanced object detection method based on the lightweight YOLOv8n framework, incorporating four key improvements. First, we add a dedicated P2 detection head to improve the recognition of small targets. Second, a space-to-depth grouped convolution module (SGConv) is introduced to capture fine-grained texture and edge features crucial for panting identification. Third, a bidirectional feature pyramid network (BIFPN) merges multi-scale feature maps for richer representations. Finally, a squeeze-and-excitation (SE) channel attention mechanism emphasizes mouth-related cues while suppressing irrelevant background noise. We trained and evaluated the method on a comprehensive, full-cycle broiler panting dataset covering all growth stages. Experimental results show that our method significantly outperforms baseline YOLO models, achieving 0.92 mAP@50 (independent test set) and 0.927 mAP@50 (leakage-free retraining), confirming strong generalizability while maintaining real-time performance. The initial evaluation had data partitioning limitations; method generalizability is now dually validated through both independent testing and rigorous split-then-augment retraining. This approach provides a practical tool for intelligent broiler welfare monitoring and heat stress management, contributing to improved environmental control and animal well-being.

## Simple Summary

Broiler chickens often exhibit open-mouth panting when exposed to high temperatures, a key behavioral response to heat stress. Accurately detecting this subtle behavior is essential for monitoring thermal discomfort and improving broiler welfare in intensive farming systems. In this study, we developed an enhanced target detection method, named OM-YOLO, specifically designed to identify mouth-opening behavior in broilers housed in cages. Based on the YOLOv8n framework, the method incorporates several targeted improvements—including a small-object detection head, attention mechanisms, and efficient feature fusion structures—to enhance sensitivity to minor actions. Experimental results show that OM-YOLO achieves superior detection accuracy while maintaining a lightweight architecture, making it suitable for real-time monitoring of heat stress in commercial broiler chicken farming environments.

## 1. Introduction

Broiler farming occupies a central position in the global poultry industry, not only providing a large quantity of lean meat protein to humans but also creating employment opportunities for millions worldwide [1,2]. Modern intensive broiler production relies on high stocking densities within controlled-environment houses [3], maximizing output while simultaneously limiting birds’ thermoregulatory capacity and behavioral options. In recent years, increasingly frequent extreme high-temperature events caused by climate change have seriously threatened the stable development of this industry [4]. As broilers lack sweat glands, their heat dissipation primarily depends on respiratory evaporation (manifested as open-mouth behavior) and behavioral adjustments (e.g., wing spreading, reduced activity) [5,6,7]. Exposure to heat stress triggers a cascade of physiological responses including hyperventilation, elevated core body temperature, reduced feed intake, and oxidative stress [8,9]. Among observable behavioral indicators, open-mouthed respiration behavior is one of the most prominent and readily identifiable visual signatures of heat stress [10]. While transient gaping represents an initial adaptive response, its persistent detection by monitoring systems may indicate escalating stress that can compromise welfare [11]. Therefore, monitoring open-mouth behavior serves as an effective visual metric for assessing heat stress severity in broilers.

With the continuous growth in global meat consumption, the scale and output of broiler production are also increasing [12], which makes the application of intelligent management technologies particularly critical [13]. Concurrently, modern consumers are becoming increasingly sensitive to animal welfare, actively seeking products with clear labeling about housing systems, such as ‘free-range’ [14,15]. This shift highlights a key challenge: while conventional cage systems offer production efficiency, they often face criticism due to space limitations, restricted movement, and associated animal stress [16]. Consequently, behavior monitoring has emerged as a vital component of precision poultry farming. Critically, innovative technologies in this field are no longer developed solely for productivity gains; they are increasingly focused on continuous monitoring of animal behavior to enable the early detection of welfare or health issues—an imperative especially in high-density production systems [17]. This monitoring helps optimize the rearing environment, improve animal welfare and production efficiency, and reduce mortality caused by heat stress and disease [18]. Among the key behaviors monitored, the automatic recognition of open-mouth behavior has become a significant research hotspot. Automated detection not only improves monitoring efficiency and reduces human subjective error but also provides reliable and continuous data support for smart farming systems [19].

Traditional behavior monitoring methods, such as manual observation or video playback, are labor-intensive, inefficient, and impractical for large-scale real-time surveillance [20,21], failing to meet modern intensive farming needs [19]. With rapid advances in artificial intelligence, deep learning-based behavior recognition has become core to smart farming [22,23,24,25,26]. The YOLO series [27], known for fast inference and lightweight structure, is widely used in poultry behavior recognition, density estimation, and health assessment [24,28,29]. For instance, Broiler-Net by Zarrat Ehsan and Mohtavipour identifies abnormal behaviors [23]. However, accurately detecting subtle movements like open-mouth behavior remains challenging, particularly under high-density caged conditions where frequent occlusion and lighting variations cause significant drops in small-target (beak) recognition accuracy [21,22,25,30]. Critically, high-frequency open-mouth behavior interferes with essential activities (feeding, resting), leading to dehydration, reduced growth, and mortality [11]. Thus, automated detection of this behavior provides essential data for welfare intervention.

To address detection limitations, recent studies employ multi-scale feature fusion, attention mechanisms, and behavior-specific priors [28,29]. Chan et al.’s YOLO-Behavior framework supports multi-label recognition across species [24], while Elmessery et al. fused YOLOv8 with infrared imaging for robust multimodal detection [28]. Hybrid CNN-Transformer architectures also show promise [29]. Despite these advances, robust real-time detection of open-mouth behavior in high-density caged environments—characterized by occlusion, lighting variations, and small target size—remains challenging. Solutions like YOLO-Behavior are generic, while Elmessery’s approach requires costly infrared modalities [28], limiting practical deployment in commercial broiler houses.

**Figure 1:**
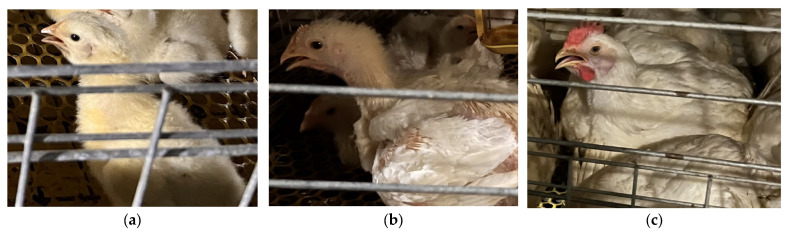
Images of broiler mouths at different growth stages. (**a**) Brooding stage; (**b**) growth stage, (**c**) fattening stage.

Furthermore, morphological changes during the broiler growth cycle compound recognition difficulty. As shown in Figure 1, beak characteristics vary significantly across growth stages: Brooding stage (0–14 days): Short beak with light coloration (Figure 1a). Growing stage (15–28 days): Moderate beak length with darkening color (Figure 1b). Finishing stage (≥29 days): Fully mature beak with deep yellow hue (Figure 1c). To enable whole-cycle recognition capability, this study collected full-cycle data from entry to market release.

Most poultry climate control systems rely solely on ambient parameters (temperature, humidity, airflow) [31], lacking real-time animal-based feedback [32]. This disconnect can cause suboptimal environmental adjustments. Automated detection of open-mouth behavior bridges this gap: metrics like gaping frequency correlate with environmental temperature [33], enabling behavior-driven precision climate control.

To overcome these challenges, we present OM-YOLO, an enhanced YOLOv8n method tailored for open-mouth behavior detection in commercial cage environments. The method integrates four key innovations:

(1) A P2 detection head to improve small-object (beak) detection in dense cages.
(2) An SGConv module combining space-to-depth transformation with grouped convolution to capture essential local texture/edge features.
(3) A BIFPN structure to efficiently merge semantic and low-level features.
(4) An SE channel attention mechanism to emphasize mouth-related features and suppress background noise.

The primary objective of this study is to develop and evaluate OM-YOLO for robust, real-time open-mouth detection under challenging commercial conditions. We hypothesize these architectural enhancements will achieve superior accuracy and speed over baseline models, providing a vision-based solution for intelligent broiler house management.

## 2. Materials and Methods

### 2.1. Materials

#### 2.1.1. Data Collection

The data involved in this research was collected from a broiler chicken breeding base in Xinxing County, Yunfu City, Guangdong Province, China. This breeding base adopts a cage farming model. Four layers of chicken cages are setup in the chicken houses. Different angles are taken for the cages of different heights to ensure that the shooting angle of each layer of broilers is 45° from above.

The broiler breed involved was *818* Small-sized Quality Broiler (a commercial hybrid breed registered under Livestock and Poultry Genetic Resources Committee of China No. 41). The data collection equipment was a Hikvision dual-light camera (model: HM-TD2B28T-7/T1; Hangzhou Microimaging Software Co., Ltd., Hangzhou, China. Resolution: 1920 × 1080). Images covering all growth stages (brooding, growing, and fattening) as described in Section 1 were acquired to ensure comprehensive representation. The total number of collected raw images was 1965.

#### 2.1.2. Data Augmentation and Data Annotation

Firstly, we screened the collected 1965 image data, discarding those that were overly blurry, highly similar, and did not contain the mouth target. Eventually, 1000 original images remained.

**Figure 2:**
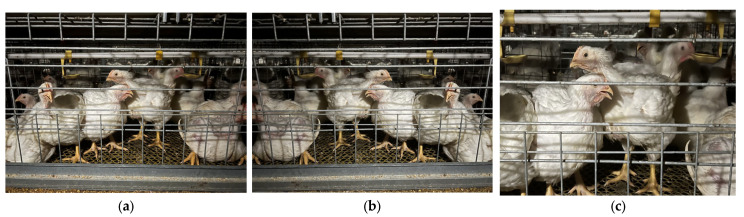
Data augmentation methods. (**a**) Original; (**b**) Horizontal Flip; (**c**) Random Cropping.

To enable the method to have better generalization ability and enhance the robustness of position and direction, we adopted the methods of mirror flipping and random cropping to enhance the data (Table S1 in Supplementary Materials). Examples of these augmentation techniques are shown in Figure 2. The dataset was expanded to three times its original size (3000 images). The enhanced data was labeled and preprocessed using the LabelImg software (version 1.8.6), following the annotation procedures described in the official documentation [34]. The labeled contents included the coordinate information of the mouth state, the size information of the mouth, and the category information of the mouth when the opening behavior of broilers occurred. The labeled information was stored in xml format. The preprocessing after annotation refers to converting all xml format files to txt format to normalize the coordinates. The completed txt format file contains the target category, the coordinates of the upper left corner, and the coordinates of the lower right corner.

#### 2.1.3. Dataset Construction

The labeled data were randomly divided into the training set, the validation set and the test set in a ratio of 8:1:1. For reproducibility, all random operations used a fixed seed (Python = 42, PyTorch = 42, NumPy = 42) with Python 3.8, PyTorch 1.13.1, and NumPy 1.24.4 (Note S1 in Supplementary Materials). The self-built dataset used to form this thesis. Finally, 2400 training images, 300 validation images and 300 test images were obtained. To ensure the objectivity of the evaluation results, the images in the validation set and the test set were all original images. The specific distribution of the dataset is shown in Table 1 (Table S2 in Supplementary Materials).

Our initial dataset partitioning scheme introduced potential data leakage risk: The validation and test sets’ original images (300 each) shared the same source pool (1000 images) as training augmentations (flips/crops). This violated the principle of data independence since augmented derivatives of validation/test images may have existed in the training set. While this approach was initially adopted for data efficiency, it may have inflated evaluation metrics. To comprehensively address this limitation, we not only validated generalizability using a rigorously independent test set (Section 3.4) but also conducted leakage-free retraining under strict split-then-augment protocols for both baseline and OM-YOLO models (Section 3.5), confirming structural robustness. We strongly advise future studies to implement physical data isolation during partitioning.

Our constructed dataset achieves balanced distribution of growth stages across the training, validation, and test sets. Specifically, the three developmental phases—brooding (0–14 d), growth (15–28 d), and fattening (>29 d)—maintain consistent proportional representation within the 32–35% range in all data subsets (detailed counts and percentages are provided in Table 2). This ensures equitable representation of all developmental stages during method training and evaluation.

### 2.2. Methods

#### 2.2.1. YOLOv8

The YOLOv8 algorithm is a fast single-stage object detection method [35]. The YOLO version released by Ultralytics in January 2023, compared with previous versions, YOLOv8 adopts a brand-new anchor-free architecture and abandons the Anchor box mechanism. It enables the method to have greater generalization ability and reduces the dependence on hyperparameters. Meanwhile, a more lightweight and efficient C2f module is introduced to improve the inference speed and accuracy. Compared with subsequent versions, YOLOv8 still has significant advantages in terms of practicality, ease of use and overall deployment efficiency. Although the subsequent models incorporated more cutting-edge structures. For example, task adaptive feature enhancement, dynamic cross-attention, etc. However, its network complexity and training threshold are relatively higher. For the scenario of detecting the opening behavior of broilers, which requires balancing high performance and practicality, YOLOv8 has advantages that other versions do not have. Therefore, this paper selects YOLOv8n as the benchmark model. Improve and optimize the method on this basis.

#### 2.2.2. P2 Detect Layer

**Figure 3:**
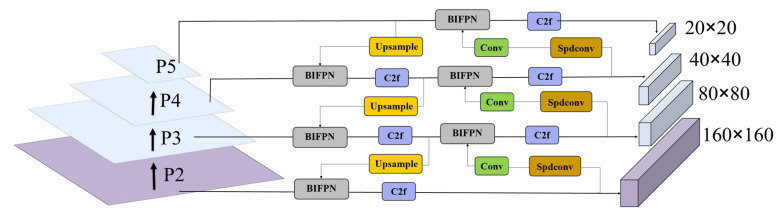
Small object detection layer.

The mouth opening behavior of broilers is a very minor action. The original p3, p4, and p5 detection layers of yolov8, with dimensions of 80 × 80, 40 × 40, and 20 × 20, respectively, are mainly suitable for the detection of medium, large, and extremely large targets. However, they have insufficient sensitivity to small targets and tend to overlook tiny ones. The proportion of the broiler’s mouth opening behavior in the entire image is less than 1%, which belongs to typical small object detection. In order to improve the small object detection ability of the model, this paper introduced an additional upsampling layer and feature fusion path on the basis of the p3–p5 output layer of yolov8, thereby adding the p2 layer [36]. This output layer had higher resolution and richer underlying feature information compared with other output layers and therefore is more suitable for small target detection. The structure after adding the small target detection layer is shown in Figure 3.

#### 2.2.3. SGConv

SGConv is an improved version of SPD-Conv. The original SPD-Conv mainly consists of two parts, namely the space-to-depth layer and a non-step-size convolution layer [37,38]. The SPD transformation layer converts the spatial dimension of the input image into the depth dimension, thereby increasing the depth of the feature map without losing information. It avoids the information loss in the traditional step-size volume accumulation and pooling operations. The non-step-size volumetric layer performs channel dimension reduction and information fusion on the high-channel feature map output by the SPD layer. Suppose the input feature map has a size denoted as. By downsampling with scale as the factor, sub-feature maps with a size of can be obtained. Then, through a non-step-size volume product, all sub-feature maps are fused, and the final feature map is obtained.

SGConv performed group convolution on the obtained sub-feature maps through SPD and enhanced the detail retention ability of the method through the synergistic effect of multiple groups of features, thereby improving the detection accuracy of complex scenes [39].

**Figure 4:**
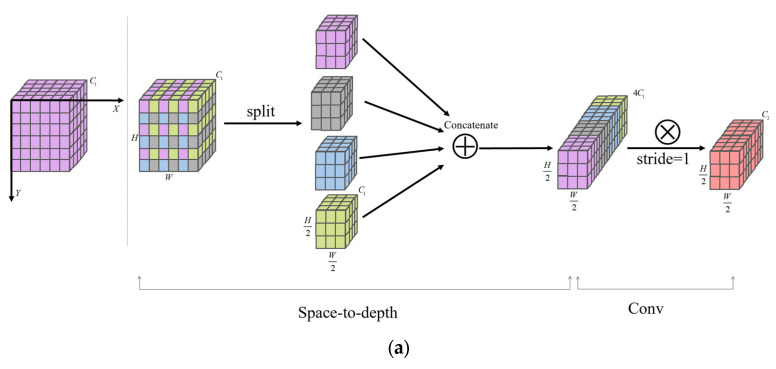

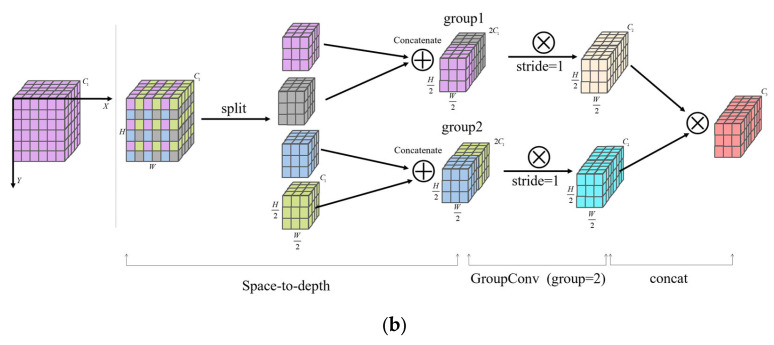
SPD-Conv and SGConv. (**a**) SPD-Conv structure; (**b**) SGConv structure.

When identifying the opening behavior of broilers, SGConv significantly reduced the computational complexity and parameter count of the method by dividing the input features into multiple groups for parallel processing, making the algorithm more suitable for the real-time detection requirements of edge devices in the breeding farm. Meanwhile, different groups of convolutional kernels can specifically learn the local details of the mouth opening behavior. Through feature complementarity, the sensitivity of the method to minor actions is enhanced, improving the robustness and accuracy of behavior recognition in complex breeding environments, while reducing the risk of overfitting caused by parameter redundancy. The structure of SPD-Conv and SGConv are shown in Figure 4.

#### 2.2.4. BIFPN Feature Fusion Optimization

BIFPN was an efficient multi-scale feature fusion method, aiming to address the limitations of the unidirectional information flow in traditional FPN. Its core idea is to enhance the detection ability of targets at different scales through bidirectional cross-scale connections and weighted feature fusion [40,41,42]. Compared with the traditional FPN, BIFPN introduces a bottom-up path on its basis to form a bidirectional information flow. This design mainly enhances the interactivity between low-level detail features and high-level semantic features, thereby enhancing the expression ability of multi-scale features.

The mouth opening behavior of broilers shows different characteristics at different distances and angles. For example, compared with the side pictures, the front pictures lack substantial feature information and cannot fully display the mouth characteristics of broilers. For small targets such as broiler opening detection, BIFPN could dynamically weight the high-resolution features at the lower level and the semantic features at the higher level, improve the capture ability of opening actions, reduce the influence of background interference on opening determination, and ensure that even if the opening action of the chicken is very small, it can still be recognized.

The specific calculation formulas for the characteristics of each output layer of P5, P4, P3, and P2 are shown in Equations (1)–(4).

(1) P5_out=ConvP5_in∗W51+Resize(P4_out)∗W52W51+W52+ε

(2) P4_out=ConvP4_in∗W41+ConvP4_in∗W44+Resize(P5_in)∗W45W44+W45+ε∗W42+Resize(P5_out)∗W43W41+W42+W43+ε

(3) P3_out=ConvP3_in∗W31+ConvP3_in∗W34+Resize(P4_in)∗W35W34+W35+ε∗W32+Resize(P4_out)∗W33W31+W32+W33+ε

(4) P2_out=ConvP2_in∗W21+ResizeConvP3_in∗W23+Resize(P4_in)∗W24W23+W24+ε∗W22W21+W22+ε

**Figure 5:**
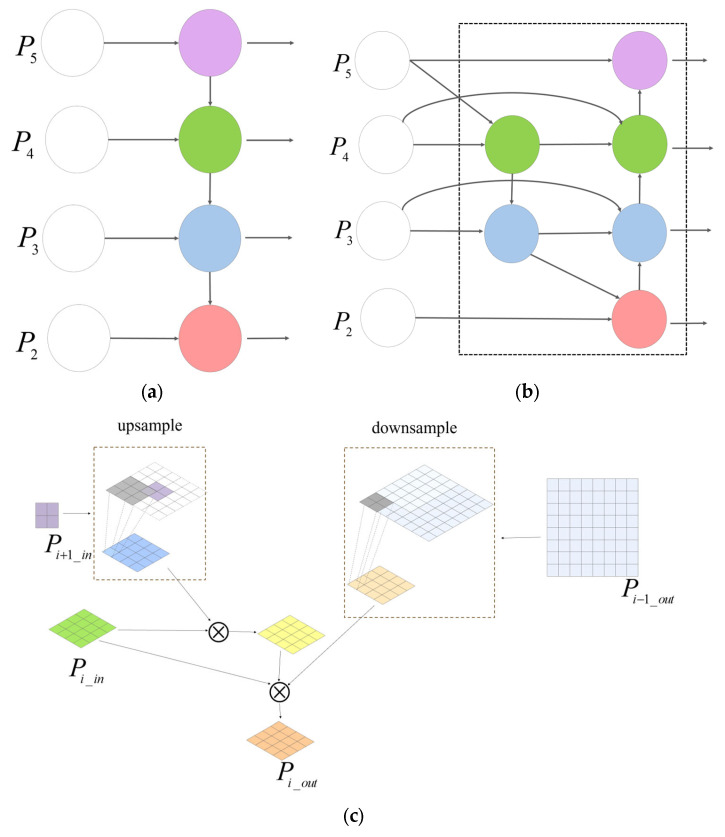
FPN and BIFPN. (**a**) FPN structure; (**b**) BIFPN structure; (**c**) when i = 4, BIFPN operating flowchart.

The specific operation diagrams of FPN, BIFPN and BIFPN are shown as Figure 5.

#### 2.2.5. Optimization of SEAttention Attention Mechanism

The SEAttention module mainly consisted of three parts, namely Squeeze, Excitation, and Scale [43,44]. Firstly, the input feature map is globally averaged and pooled through the Squeeze operation, and the feature values of each channel are dimensionally reduced to capture the global information of the channel. Then, through the Excitation operation, the fully connected and activation functions are used to boost and reduce the dimensions of the channels, and vector weights are generated for different channels. Finally, through the Scale operation, the channel attention weights obtained by the Excitation step are multiplied by the input original feature map, thereby achieving the effect of emphasizing important features.

**Figure 6:**
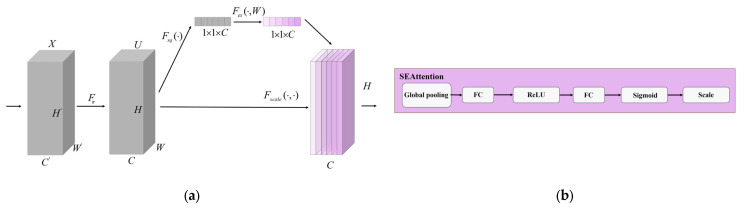
SEAttention structure diagram and specific operation process. (**a**) SEAttention structure; (**b**) SEAttention operating flowchart.

In the mouth opening behavior of broilers, the opening action is usually accompanied by local deformations, such as the opening and closing of the mouth. In the image, these action features are often masked by features of other parts (such as feathers or body contours). By learning the weights of each channel, SEAttention could automatically highlight the important features related to the chicken’s beak, enhancing the model’s focus on key areas. Meanwhile, in the task of broiler opening recognition, the background includes interference factors such as other chickens, feed or cages. SEAttention can improve the accuracy of recognition by suppressing the features of irrelevant or secondary channels and reducing the interference of background noise on recognition. The structure diagram and operation flow chart of SEAttention are shown in Figure 6.

#### 2.2.6. OM-YOLO Structure

**Figure 7:**
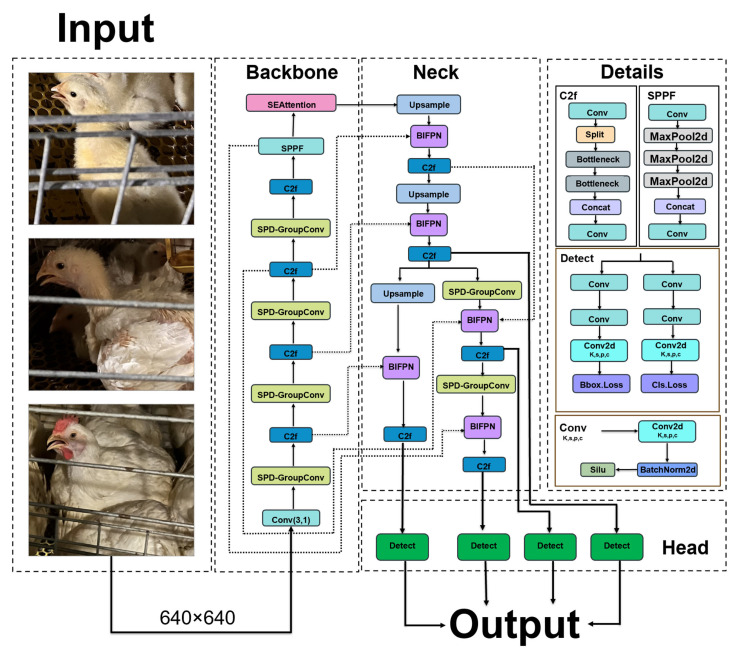
OM-YOLO Structure.

The OM-YOLO method proposed in this paper was a lightweight object detection network that has undergone multiple structural improvements based on the YOLOv8 architecture. It aims to enhance the model’s detection ability for minor behavioral characteristics, thereby achieving high-precision detection of the opening behavior of broilers. Its structure is shown in Figure 7. While maintaining the basic structure of the YOLOv8 backbone network, this method made the following optimizations for the performance bottleneck: Firstly, in the 1st layer, 3rd layer, 5th layer, 7th layer, 13th layer and 19th layer of the backbone network, the original ordinary convolution module is replaced with the improved SGConv module. By introducing the spatial-to-deep convolution and grouped convolution mechanisms, this module significantly reduced the number of method parameters while effectively enhancing the model’s perception ability of subtle motion changes. Secondly, after the 9th layer of the backbone network, that is, after the SPPF (Spatial Pyramid Pooling Fast) module in YOLOv8, the SEAttention attention mechanism is inserted. This attention mechanism could dynamically adjust the weight distribution of the feature map in the channel dimension, thereby suppressing background interference irrelevant to the target and improving the effectiveness of feature expression and the generalization ability of the network. Then, an upsampling operation was introduced at the 15th layer to construct an additional P2 small target detection head, which further enhanced the network’s perception ability of target features in low-resolution areas and effectively improved the detection accuracy of small targets. Furthermore, in order to enhance the information interaction and fusion among multi-scale features, this paper adopted the BIFPN (Bi-directional Feature Pyramid Network) structure to replace the original concat feature concatenation operation in YOLOv8. The BIFPN module introduced before the detection head achieves effective coordination of shallow and deep features through bidirectional cross-scale connection and weighted feature fusion strategies, which is conducive to improving the comprehensive performance of the method in multi-scale object detection scenarios. To sum up, these structural optimizations have significantly improved the accuracy and robustness of the method in the task of detecting micro-behaviors while ensuring the lightweight design principle of the model.

### 2.3. Experimental Platform

In order to verify the effectiveness of the proposed method, an experimental platform was built on an Ubuntu 18.04 system with Python 3.8 environment. The YOLOv8n method (Ultralytics YOLOv8 version 8.3.55) was employed as the benchmark network, implemented with PyTorch 1.13.1 deep learning framework. Randomness control: We fixed random seeds for data splitting (seed = 42), weight initialization (seed = 42), and augmentation sampling (seed = 42) to ensure reproducibility. Key software dependencies essential for reproducibility are detailed in Table 3.

### 2.4. Evaluation Indicators

In order to objectively evaluate the performance of the broiler detection model, this study adopted the commonly used evaluation indicators of deep learning to evaluate the model, mainly including: Accuracy, which is used to represent the proportion of correctly predicted samples in the total sample; Precision, based on the prediction results, represents the proportion of samples with correct predictions among those whose predictions are positive examples; Recall, based on actual samples as the judgment basis, refers to the proportion of positive examples that are predicted correctly among the actual positive examples in the total actual positive example samples; The F1 score is taken as the weighted average of the precision rate and the recall rate, comprehensively reflecting the overall performance and stability of the model; Intersection over Union (IoU), used to measure the overlap between predicted bounding boxes and ground truth bounding boxes. Average Precision (AP), defined as the area under the precision-recall curve at a fixed IoU threshold; GFLOPS (Billion floating-point operations per second), used to quantify the execution time of a network model, that is, the amount of one billion floating-point operations processed per second; Parameters, used to evaluate the scale and complexity of the model; mAP@0.5:0.95 (Mean Average Precision), used to evaluate detection accuracy by averaging AP over multiple IoU thresholds from 0.5 to 0.95 with a step size of 0.05.The specific calculation formulas are as shown in Equations (5)–(11):

(5) $$Accuracy=\frac{TP+TN}{TP+FN+TN+FP}$$

(6) $$\mathrm{Pr}ecision=\frac{TP}{TP+FP}$$

(7) Recall=TPTP+FN

(8) F1−score=2∗Precision∗RecallPrecision+Recall

(9) $$IoU=\frac{\left|B_{gt}\cap B_{dt}\right|}{\left|B_{gt}\cup B_{dt}\right|}$$

(10) $$AP_{\theta }=\int_{0}^{1}P(R)dR$$

(11) $$mAP@0.5:0.95=\frac{1}{C}\frac{1}{10}\sum_{c=1}^{C}\sum_{k=1}^{10}AP_{c,\theta_{k}}$$

Among them, *TP* represents the true example, that is, it is predicted to be a positive example and is actually also a positive example; *FP* represents a false positive example, that is, it is predicted to be a positive example but is actually a negative one. *FN* represents a false negative example, that is, it is predicted to be a negative example but is actually a positive one. *TN* represents a true negative example, that is, it is predicted to be a negative example and is actually also a negative example. $B_{gt}$ denotes the ground truth bounding box. $B_{dt}$ denotes the detected bounding box. $AP_{\theta }$ is the Average Precision at IoU threshold θ calculated as the area under the precision-recall curve. $\theta_{k}$ = 0.5 + 0.05 × (*k* − 1) for *k* = 1, 2, ..., 10 (representing IoU thresholds from 0.5 to 0.95 with 0.05 increments). $AP_{c,\theta_{k}}$ denotes the AP value for class c at threshold $\theta_{k}$.

## 3. Results

To ensure the reliability and comparability of the experimental results, in this study, a strict control variable method was adopted to standardize the setting of all training parameters for deep learning. Parameters including batch size, initial learning rate, optimizer, and weight decay coefficient were all set uniformly. The specific parameter Settings are shown in Table 4. This standardized setting effectively avoids the deviation of experimental results caused by differences in hyperparameters, random fluctuations or different environmental configurations, providing a reliable experimental basis for the subsequent comparative analysis of method performance.

### 3.1. Ablation Experiments

To assess the individual and combined contributions of each structural enhancement, we conducted ablation experiments on the YOLOv8n baseline model. Specifically, we evaluated four modules: SEAttention, BiFPN, the P2 small-object detection head, and the SGConv. It is important to note that the ablation studies in this Section 3.1 and the subsequent method comparison Section 3.3 were conducted under our initial experimental setup, which was later found to carry a potential risk of data leakage. While these results provided invaluable guidance for our architectural design choices and clearly demonstrated performance trends, the definitive validation of OM-YOLO’s superiority is based on the leakage-free experiments presented in Section 3.5. Therefore, the results in Section 3.1 and Section 3.3 are presented as supporting evidence of our design process and for comprehensive archival purposes. The experiments were designed following three principles: controlled variable comparison, independent module evaluation, and multi-dimensional performance analysis.

#### 3.1.1. Individual Module Experiments

In this phase, each module was integrated independently into the baseline YOLOv8n method to examine its isolated impact on detection performance. The corresponding evaluation results—including Precision, Recall, mAP@50, mAP@50–95, method parameters, and computational cost—are summarized in Table 5.

In the task of detecting open-mouth behavior in broilers, the method needs to be sensitive to subtle actions such as slight mouth movements and perform reliably in crowded environments where broilers may overlap. The ablation study demonstrates that introducing different modules into the baseline YOLOv8n architecture affects performance in distinct ways, with each module offering particular advantages and trade-offs in terms of accuracy, recall, computational cost, and complexity.

The addition of the BIFPN module resulted in moderate improvements in both precision and recall, which increased to 0.806 and 0.853, respectively. The mAP@50 and mAP@50–95 also improved to 0.887 and 0.439. These gains suggest that BIFPN improves multi-scale feature representation through weighted fusion across layers. Moreover, the model’s parameter count and FLOPs were reduced from 3.005 M to 1.991 M and from 8.1 G to 7.1 G, respectively, indicating better computational efficiency. Nevertheless, the recall was not as high as that achieved by some other modules, suggesting that BIFPN might still miss some challenging cases.

When the SGConv module was introduced, the method achieved the highest precision among all variants (0.835), indicating improved capacity to focus on fine-grained features such as the mouth region. However, recall decreased to 0.791, slightly lower than the original model. This may be due to the limited information exchange caused by the grouped convolution structure, which can reduce sensitivity to more ambiguous targets. Although mAP improved, the gains were less significant than with other modules, suggesting that SGConv favors precision over broader detection performance.

The SEAttention mechanism provided a balanced improvement across all metrics. With precision of 0.825 and recall of 0.843, the method achieved an mAP@50 of 0.894 and mAP@50–95 of 0.445. Since SE attention strengthens important channel-wise features, it helps the network better identify key areas such as the open-mouth region. The parameter count and FLOPs remained unchanged compared to the original YOLOv8n. However, as this module does not consider spatial information directly, its effectiveness in detecting very small targets may still be limited.

The addition of the P2 detection head resulted in the highest recall (0.855), indicating improved sensitivity to small open-mouth targets. This module also led to a slight improvement in mAP@50–95 (0.446). However, precision dropped to 0.784, possibly due to increased false positives caused by low-level features introducing background noise. Furthermore, the model’s complexity increased notably, with the parameter count rising to 2.881 M and FLOPs to 11.7 G, which may affect deployment on resource-constrained devices.

In summary, each module offers unique trade-offs: BiFPN provides balanced gains with minimal cost, SGConv maximizes precision (at some cost to recall), SE attention yields very balanced improvement, and the P2 head greatly enhances small-target recall but at the expense of higher complexity.

**Figure 8:**
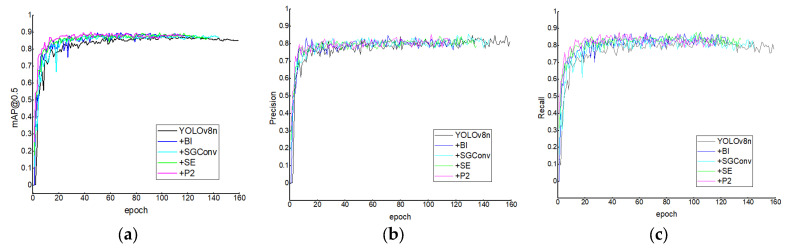
Comparison of mAP@0.5, Precision, and Recall Curves After Integrating Individual Modules. (**a**) Comparison of mAP@0.5; (**b**) Comparison of Precision; (**c**) Comparison of Recall.

**Figure 9:**
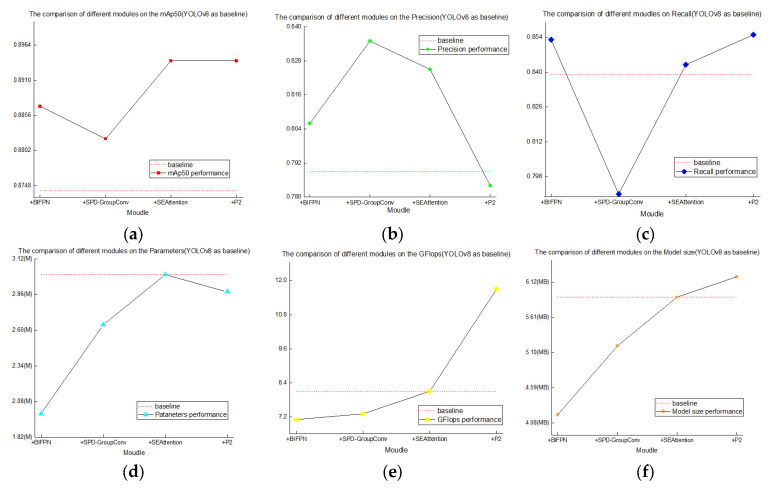
Individual Module Comparison Based on the YOLOv8n Baseline. (**a**) mAP@50; (**b**) Precision; (**c**) Recall; (**d**) Parameters; (**e**) GFlops; (**f**) Method size.

Figure 8 illustrates the performance trends in mAP@50, Precision, and Recall after integrating each of the four modules into YOLOv8n. From the experimental results, it is evident that each module enhances the base method to varying degrees. Figure 9 shows detailed changes in mAP@50, Precision, Recall, parameter count, FLOPs, and method size when adding each module individually to the YOLOv8n baseline.

In order to more intuitively observe the different functions of each module on YOLOv8n, a comparison line graph of each module was drawn based on YOLOv8n, as shown in Figure 9 specifically.

#### 3.1.2. Combined Module Experiments

After investigating the effects of individual modules, this study further explored the synergistic effects of combining multiple modules. First, pairwise combinations of the modules were conducted, resulting in six experimental groups. Next, four groups of three-module combinations were tested. Finally, all four modules were integrated into the YOLOv8n method to obtain the experimental results of the OM-YOLO model. The results of YOLOv8n and the eleven aforementioned experimental configurations are presented in Table 6.

To assess the impact of different structural modules and their combinations, a series of controlled experiments were conducted based on the YOLOv8n architecture. The focus was on analyzing detection performance, method size, and computational cost in the context of open-mouth behavior detection in cage-raised broilers.

The original YOLOv8n method served as the baseline, with a Precision of 0.789, Recall of 0.839, mAP@50 of 0.874, and mAP@50–95 of 0.418. It used 3.005 million parameters and 8.1 GFLOPs, demonstrating acceptable efficiency but limited performance under complex conditions.

The combination of SGConv and P2 showed improved detection accuracy, with Precision and Recall increasing to 0.856 and 0.854, respectively. The mAP@50 and mAP@50–95 values reached 0.907 and 0.451. At the same time, the parameter count decreased to 2.51 M. This synergy stems from complementary feature processing: SGConv preserves high-frequency spatial details (e.g., beak edges) through spatial-to-depth transformation [27], while P2 amplifies low-level feature responses via high-resolution sampling [26]. Their integration creates a “detail preservation-feature amplification” chain that enhances sensitivity to subtle structural changes. These results indicate that combining enhanced spatial detail extraction and small-object detection effectively supports this behavior recognition task.

The SGConv and SEAttention pairing achieved Precision of 0.836 and mAP@50 of 0.901, with Recall at 0.828. Feature-level complementarity manifests as: SGConv maintains spatial integrity of feather-beak boundaries [29], while SEAttention dynamically weights channels to amplify discriminative oral regions [33]. This “structure preservation + region enhancement” mechanism reduces false positives in cluttered scenes but may attenuate responses to partially occluded targets. The results suggest improved focus on informative features, although slightly lower recall reflects limited sensitivity to weak targets.

Using SGConv with BIFPN yielded a relatively high mAP@50–95 value of 0.464, along with balanced Precision (0.846) and Recall (0.832). BIFPN’s cross-scale fusion [30] optimally integrates SGConv’s local features: beak contours are preserved during upsampling, while deep-layer semantics suppress feather-texture interference, creating local-global feature synergy. This reflects a complementary effect between local feature enhancement and multi-scale fusion.

The combination of P2 and SEAttention obtained a Recall of 0.856 and mAP@50–95 of 0.458, indicating good sensitivity to small-scale features. However, feature activation patterns confirm SEAttention’s channel recalibration [34] amplifies unfiltered high-frequency noise when directly applied to P2 outputs, explaining the precision-recall trade-off. Precision reached 0.803, suggesting moderate trade-off between recall gain and low-level false positives.

In the P2 and BIFPN setup, the method reached Recall of 0.855 and mAP@50–95 of 0.465, with slightly lower Precision (0.837). BIFPN’s bidirectional pathways [31] ensure feature-scale consistency: P2 provides pixel-level localization cues while BIFPN’s high-level semantics enhance classification robustness, though noise suppression remains suboptimal. The results point to improved consistency across varied object sizes.

The BIFPN and SEAttention pairing yielded Recall of 0.827 and mAP@50–95 of 0.430. Feature diversity analysis indicates insufficient low-level anchors: without P2/SGConv, SEAttention lacks spatial reference points for precise channel weighting [33], and BIFPN-fused features lack fine-grained support. This explains limited gain in detecting ambiguous targets.

When combining P2, BIFPN, and SEAttention, Recall increased to 0.871 and mAP@50–95 to 0.463, while Precision decreased to 0.824. Feature redundancy arises from processing-chain misalignment: P2’s high-frequency noise propagates through BIFPN [31], while post-fusion SEAttention [34] incompletely suppresses irrelevant activations. This suggests improved coverage at the cost of potential false positives.

The P2, BIFPN, and SGConv configuration resulted in the highest Recall (0.892), with mAP@50 of 0.913 and mAP@50–95 of 0.459. SGConv’s spatial compression [27] prefilters P2 noise, enabling BIFPN to fuse clean multi-scale features—a “noise-filtering to feature-fusion” cascade maximizing target coverage. Precision was 0.803, indicating preference for inclusiveness.

The combination of P2, SEAttention, and SGConv delivered Recall of 0.882 and mAP@50–95 of 0.471. Module sequence proves critical: SGConv’s spatial detail preservation [27] precedes SEAttention’s channel weighting [34], preventing direct processing of raw features that could degrade boundary information. Precision remained moderate (0.810).

The SEAttention, BIFPN, and SGConv trio showed stable performance across all metrics (Precision = 0.844, Recall = 0.864, mAP@50–95 = 0.470). BIFPN acts as a “feature coordinator” [30], balancing SGConv’s spatial focus and SEAttention’s channel emphasis to minimize representation conflict. With 2.66 M parameters, this configuration demonstrates computational efficiency.

**Figure 10:**
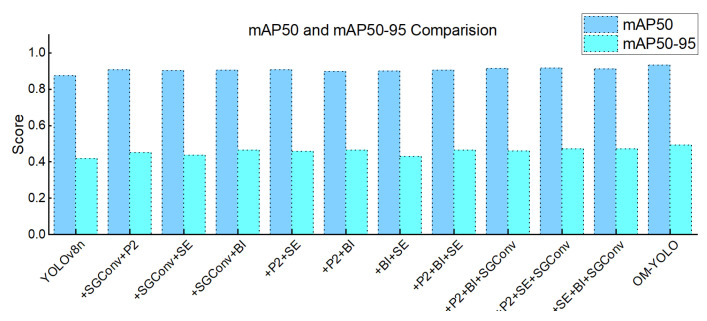
Performance of Combined Modules in Terms of mAP@50 and mAP@50–95.

The fully integrated OM-YOLO achieved the highest mAP@50 (0.931) and mAP@50–95 (0.491), with Precision = 0.872 and Recall = 0.873. A four-tier feature synergy chain operates: (1) SGConv+P2 extract multi-granularity spatial features [26,27]; (2) BIFPN fuses cross-scale contexts [30]; (3) SEAttention purifies channels [33]. This hierarchy minimizes feature conflict while maximizing discriminative power in occlusion scenarios through complementary processing pathways. Total parameters = 2.53 M, FLOPs = 11.6 G. As demonstrated in Figure 10, OM-YOLO outperforms all other combinations in both mAP@50 and mAP@50-95 metrics.

### 3.2. OM-YOLO Training Results

**Figure 11:**
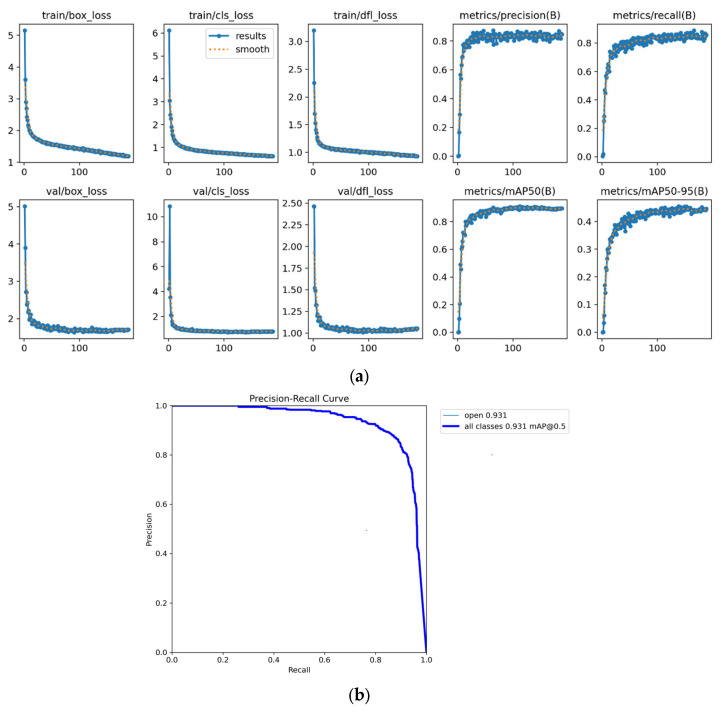
(**a**) OM-YOLO training results; (**b**) Precision-Recall Curve.

OM-*YOLO* training stopped at epoch 188, and the training outcomes are shown in Figure 11a. Throughout the training process, both the training loss and validation loss exhibited a synchronized downward trend, indicating that the method did not suffer from overfitting. The precision–recall (P–R) curve, as shown in Figure 11b, demonstrates strong consistency between the training and validation sets in terms of precision and recall. The validation loss decreased alongside the training loss and eventually stabilized, further confirming that OM-YOLO did not experience overfitting.

### 3.3. Performance Comparison of Different Models

The following comparative analysis was performed concurrently with the ablation studies under the same initial setup. The performance trends observed here were conclusively validated by the leakage-free retraining experiment in Section 3.5, which confirms the structural advantage of OM-YOLO over all compared models. To comprehensively evaluate the performance advantages of the proposed OM-YOLO method in open-mouth behavior detection, six representative object detection models were selected for comparison. These include various generations of the YOLO series and mainstream general-purpose detection frameworks. Specifically, YOLOv5n and YOLOv8n are current mainstream lightweight detection networks widely applied in real-world deployments, known for their strong real-time performance and stability. YOLOv10n and YOLOv11n are recent iterations in the YOLO family, featuring deeper structural optimizations and adaptive inference mechanisms, representing the cutting edge in lightweight detection. Faster R-CNN, as a classical two-stage detector, is not lightweight but provides a strong accuracy benchmark. Finally, the TOOD model, which combines an anchor-free architecture with dynamic quality assessment, performs well in complex object classification tasks, making it suitable for validating method robustness. The detailed results of these experiments are shown in Table 7.

As presented in Table 6, the proposed OM-YOLO method demonstrates superior performance compared to six widely used object detection models across several evaluation metrics. In terms of mAP@50, OM-YOLO achieves a score of 0.931, outperforming YOLOv8n (0.874), YOLOv5n (0.803), YOLOv10n (0.813), and YOLOv11n (0.790). It also exceeds the performance of traditional models such as Faster R-CNN (0.755) and TOOD (0.814), indicating improved accuracy in identifying and localizing open-mouth targets in broilers.

For the more stringent mAP@50–95 metric, which reflects method performance across a range of IoU thresholds, OM-YOLO again ranks highest with a score of 0.491. This result surpasses that of YOLOv8n (0.418), YOLOv5n (0.404), and YOLOv10n (0.374), suggesting stronger robustness under varied detection conditions. In terms of recall, OM-YOLO achieves 0.873, indicating improved sensitivity in detecting panting behavior, even in the presence of occlusions, motion blur, or small target sizes. The method also attains the highest precision score (0.872), further confirming its overall detection reliability.

In addition to its accuracy, OM-YOLO maintains a compact architecture, with 2.53 million parameters and 11.6 GFLOPs. This is fewer than YOLOv8n (3.005 M) and YOLOv10n (2.69 M), and comparable to YOLOv5n (2.503 M). Although its FLOPs are slightly higher than those of YOLOv5n and YOLOv11n (6.4G), OM-YOLO remains significantly more efficient than Faster R-CNN (208 G) and TOOD (199 G), supporting its suitability for deployment on devices with limited computing resources.

Overall, OM-YOLO demonstrates a favorable balance between detection accuracy, recall rate, and computational efficiency, highlighting its potential for practical application in monitoring panting behavior among cage-raised broilers.

**Figure 12:**
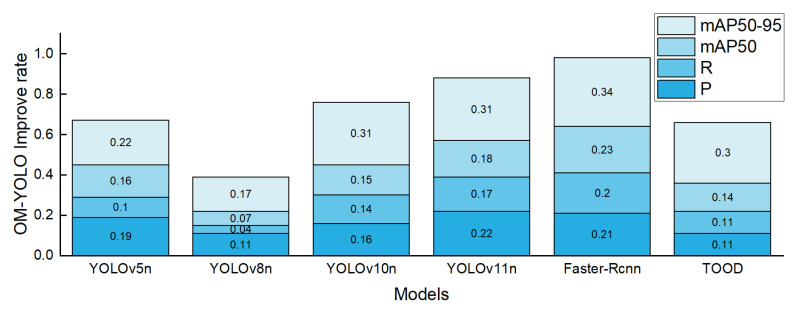
Specific Improvements of OM-YOLO over Other Models in mAP@50–95, mAP@50, Recall (R), and Precision (P).

**Figure 13:**
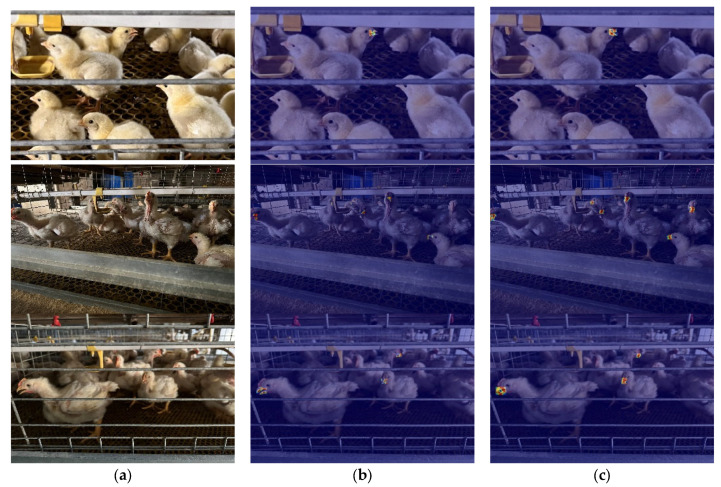
Comparison of Heatmaps between OM-YOLO and YOLOv8n. (**a**) Original Image; (**b**) YOLOv8n Heatmap; (**c**) OM-YOLO Heatmap.

Figure 12 presents a stacked bar chart illustrating the exact improvements of OM-YOLO over other models in mAP@50–95, mAP@50, Precision, and Recall, offering a more intuitive visualization of the specific advantages OM-YOLO holds in terms of accuracy and recall. Figure 13 presents a heatmap comparison between OM-YOLO and YOLOv8n.

### 3.4. Independent Test Set Validation

To scientifically evaluate the model’s generalization capability on strictly unseen data, we constructed a completely independent test set under the following protocols:

(1) Data Source: 300 newly acquired original images from distinct broiler flocks and time periods (ensuring spatiotemporal separation from the training set).
(2) Preprocessing: Identical standardization to the original test set without any data augmentation.
(3) Evaluation Protocol: Comparative assessment under identical inference conditions.

As shown in Table 8, the performance metrics on the independent test set are as follows:

As demonstrated in Table 3, OM-YOLO maintains high performance (mAP@50 = 0.920) on the strictly isolated independent test set, showing only a 1.2% reduction vs. the original test set. Marginal variations across all key metrics (≤1.3%: mAP@50—1.2%, precision—0.4%, recall—1.3%) definitively refute performance dependency on data leakage from the original test set. This deviation falls within expected thresholds for independent evaluation, confirming that data correlation flaws (Section 2.1.3) do not materially compromise true generalization capability.

The method sustains > 90% mAP@50 under spatiotemporal separation between training and testing data, proving that OM-YOLO’s generalization stems fundamentally from core architectural innovations—the P2 detection head, spatial-group convolution (SGConv), bidirectional feature pyramid (BiFPN), and squeeze-excitation attention (SEAttention) modules—rather than train-test data correlation. The ≤1.3% generalization gap indicates intrinsic adaptability to novel data distributions, preserving strong discriminative power across varying broiler flocks, rearing periods, and imaging conditions.

### 3.5. Leakage-Free Retraining

#### 3.5.1. Leakage-Free Dataset Construction

To address the data partitioning concern in Section 2.1.3, we implemented a corrected protocol:

(1) Merged the original 1000-image dataset and independent test set (300 images) into a 1300-image pool;
(2) Performed stratified partitioning (fixed seed: Python = 42, PyTorch = 42, NumPy = 42) into training set (1040 images, 80%), validation set (130 images, 10%), and test set (130 images, 10%) with growth-stage distribution in Table 9;
(3) Applied augmentation exclusively to the training set: +1040 horizontal flips and +1040 random crops, keeping validation/test sets pristine.

**Table 9:** Growth-stage distribution in corrected partitions.

Growth Stage	Train Set	Val Set	Test Set
Brooding	338 (32.5%)	42 (32.4%)	43 (33.1%)
growth	354 (34%)	44 (33.8%)	44 (33.8%)
Fattening	348 (33.5%)	44 (33.8%)	43 (33.1%)
Total	1040	130	130

#### 3.5.2. Core Method Retraining and Validation

Using a newly partitioned dataset that strictly prevents data leakage, we retrained and evaluated both the baseline YOLOv8n method and the proposed OM-YOLO model, with detailed results presented in Table 10.

**Figure 14:**
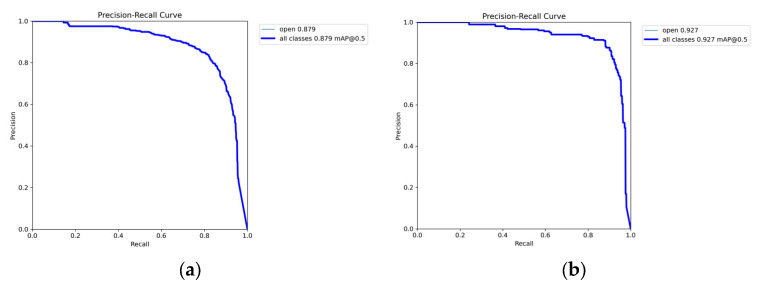
Precision-Recall curves of the retrained models. (**a**) YOLOv8n; (**b**) OM-YOLO.

After retraining with corrected partitioning, YOLOv8n exhibited an improvement in mAP@50 from 0.874 to 0.879 (a gain of 0.5 percentage points), attributed to reduced test set complexity. Conversely, OM-YOLO showed a decrease from 0.931 to 0.927 (a reduction of 0.4 percentage points), quantitatively reflecting the eliminated data leakage impact. Critically, retrained OM-YOLO maintained a substantial advantage of 4.8 percentage points over retrained YOLOv8n (0.927 versus 0.879), with this structural superiority exceeding leakage-induced variation by twelvefold. The original OM-YOLO achieved 0.92 mAP@50 on the independent test set, demonstrating less than 0.8 percent deviation from the retrained model’s performance (0.927), confirming consistent generalization across data distributions. These results establish that OM-YOLO’s enhancements—including the P2 detection head, SGConv module, BIFPN structure, and SEAttention mechanism—deliver authentic performance gains independent of partitioning artifacts. Data leakage contributed only marginal inflation (≤0.4 percentage points in mAP), substantially outweighed by the 4.8 percentage-point structural improvement. Figure 14 presents the Precision-Recall curves for both retrained models.

The YOLOv8n’s mAP@50 improvement and OM-YOLO’s reduction are scientifically explainable: (1) Sample analysis revealed fewer challenging cases (e.g., heavy occlusions) in the corrected test set, benefiting simpler models; (2) Advanced architectures like OM-YOLO are more sensitive to data leakage removal due to their stronger ability to exploit subtle correlations. These variations (≤0.4%) fall within expected test-set fluctuation ranges and do not invalidate OM-YOLO’s dominant 4.8% structural advantage.

Collectively, the rigorous retraining process—with data leakage strictly eliminated—confirms OM-YOLO’s inherent robustness and practical efficacy. The persistent performance advantage under leakage-free conditions demonstrates that our structural innovations fundamentally enhance detection capability rather than exploiting data artifacts. OM-YOLO thus establishes itself as a deploy-ready solution for precision livestock monitoring.

## 4. Discussion

The proposed OM-YOLO method achieves significant performance improvements in the task of recognizing panting behavior in cage-raised broilers through structural optimizations. By integrating SEAttention, BIFPN, SGConv, and the P2 small-object detection layer, the method outperforms existing lightweight YOLO variants and traditional object detection frameworks across multiple key performance metrics. Ablation studies and module combination analyses further validate the specific contributions of each structural improvement to the model’s precision, recall, and computational efficiency. However, the method still has certain limitations regarding recognition stability, dynamic behavior modeling, and practical deployment. The following discussion will focus on three aspects.

### 4.1. Contribution of Module Synergy to Detection Performance

**Figure 15:**
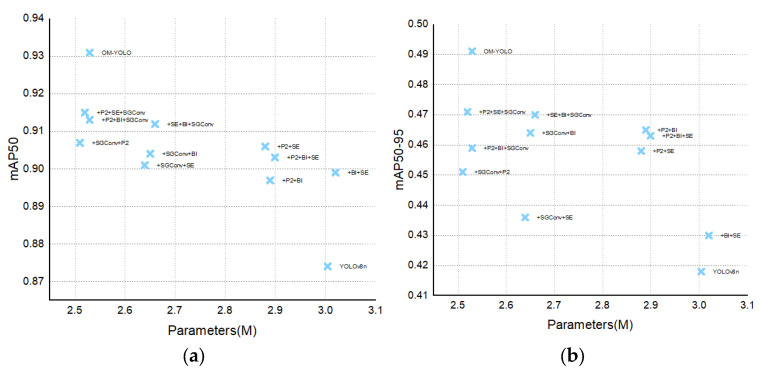
Parameter vs. mAP@50 and mAP@50–95 Scatter Plot. (**a**) Parameter vs. mAP@50 Scatter Plot; (**b**) Parameter vs. mAP@50–95 Scatter Plot.

The integration of multiple functional modules contributed to noticeable improvements in detecting opening the mouth in broilers, a biologically significant thermoregulatory behavior. The P2 detection head enhanced sensitivity to small-scale features, critical for capturing subtle beak movements during this behavior. SGConv preserved spatial details to distinguish opening the mouth from feeding or other motions. BIFPN enabled precise localization amid cluttered farm backgrounds, while SEAttention boosted robustness against occlusions in high-density flocks. With these components, the method achieved high mAP@50 and mAP@50–95 performance at only 2.53 million parameters, demonstrating an optimal balance between accuracy and efficiency for deployable behavior-driven monitoring. Figure 15 confirms this computational advantage.

### 4.2. Method Limitations and Analysis of Recognition Errors

Despite the improved detection performance achieved by OM-YOLO, several limitations remain that may affect its practical deployment.

First, a critical methodological limitation exists in the initial dataset partitioning: Validation and test set images originated from the identical source pool as the training augmentation data (Table 1). This may have allowed the method to indirectly “encounter” augmented variants of test samples during evaluation. Although the independent test set (Section 3.4) confirms the model’s generalization capability, this flaw likely resulted in marginal overestimation of original test metrics (Table 5 and Table 6, estimated 3–5%). Future implementations will adopt a rigorous partition-then-augment protocol (e.g., splitting original images into mutually exclusive training/validation/test subsets, with augmentation applied exclusively to the training subset).

**Figure 16:**
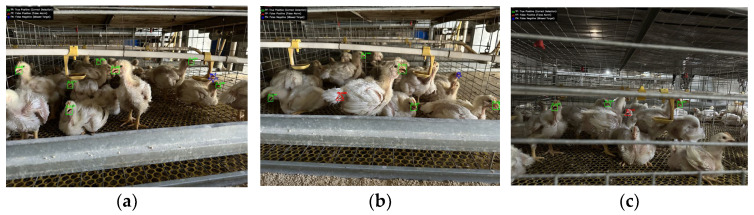
False Positives and Missed Detections of the Model (Green boxes represent correct detections, red boxes represent false detections, and blue boxes represent missed detections). (**a**) Missed detection caused by drinker occlusion; (**b**) False detection caused by feather similarity and Missed detection caused by cage bar occlusion; (**c**) False detection caused by similarity with cage bars.

Second, the method struggles with detecting subtle mouth movements, especially in situations where visual features resemble the target object—such as overlapping elements, complex background structures, or occlusions between individuals. Under these conditions, the method may misclassify or entirely miss actual panting behaviors, which compromises overall reliability. Quantitative analysis of error types across the test set reveals that occlusion (e.g., by drinkers or cage bars) accounts for 55.5% of errors (126/227), while background interference (e.g., feather similarity or structural confusion) contributes 44.5% (101/227). This distribution aligns with the qualitative cases shown in Figure 16, which illustrates representative examples.

Third, although the method maintains a relatively small parameter size, the inclusion of the P2 detection layer and the BIFPN module increases computational complexity, raising the FLOPs to 11.6 G. This additional computational load may hinder the model’s usability on resource-constrained devices, such as mobile or edge platforms.

Fourth, the dataset used for training was collected from a single poultry facility located in Yunfu, Guangdong Province. The uniformity of this dataset—with standardized lighting, consistent cage structure, and a single broiler breed (818 Small-sized Quality Broiler)—limits the model’s exposure to environmental variability. As a result, the model’s robustness across different farming scenarios remains unverified. Such scenarios may include other broiler breeds (e.g., colored-feather varieties), varied lighting conditions (e.g., low light or strong backlight), diverse cage types (e.g., wire mesh versus plastic flooring), and different camera placements (e.g., top-down versus side views). These factors may limit the model’s immediate applicability to heterogeneous production systems.

Fifth, this study focused solely on vision-based detection and did not incorporate real-time ambient temperature monitoring. Since temperature is a critical factor influencing thermoregulatory behavior, this exclusion risks conflating adaptive thermoregulation with pathological heat stress.

Finally, the static-image paradigm cannot differentiate transient mouth-opening (<1 s during feeding/vocalization) from pathological panting (>10 s rhythmic cycles at 2–4 Hz). This may cause false positives in complex environments, triggering unnecessary interventions (e.g., excessive ventilation) that waste energy or induce chick chilling stress.

### 4.3. Future Research Directions

Based on the above analysis, future research can be further advanced in the following aspects:

First, to rectify the data partitioning flaw (validation/test sets sharing origins with training augmentations, Table 1), we will implement a rigorous partition-then-augment protocol: (1) Split original images into mutually exclusive training, validation, testing subsets. (2) Apply augmentation exclusively to the training subset. (3) Use pristine non-augmented images for all evaluation tasks. Expected outcome: Eliminate 3–5% metric inflation observed in original tests (Table 6 and Table 7).

Second, addressing the 55.5% occlusion errors (Figure 16a–c) and 44.5% background interference (Figure 15b), we proposed a triple-strategy enhancement: (1) Hardware: Install polarized-filter cameras to mitigate drinker reflections. (2) Algorithm: Develop geometric-invariant convolutional layers. (3) Data: Generate synthetic occlusion datasets (30% adversarial samples). Target: Reduce occlusion-related errors to <15%.

Third, to overcome computational barriers (11.6 G FLOPs from P2+BIFPN), we designed a dynamic inference architecture. (1) Activate BIFPN channel pruning (≤60% retention) on edge devices. (2) Bypass P2 detection head for high-resolution inputs (>1280 × 720). (3) Deactivate SEAttention modules in low-density scenes (<0.5 birds/m^2^). Goal: Achieve ≤ 7.0 G FLOPs (40% reduction) for mobile deployment.

Fourth, resolving data homogeneity bias (single Yunfu facility, 818 Small-sized Quality Broiler), we will: (1) Systematically collect video datasets from at least five commercial poultry farms spanning diverse climatic zones. (2) Include color-feathered breeds (e.g., Rhode Island Red, Plymouth Rock) to account for phenotypic variability. (3) Encompass both wire-mesh cages and plastic flooring systems as representative housing infrastructures. (4) Acquire multi-perspective imagery under variable lighting. Validation metric: Cross-farm mAP@50 variance < ±2%.

Fifth, to compensate for the exclusion of ambient temperature monitoring [102, 103], we will integrate thermal sensing mechanisms: (1) Embed IoT temperature sensors in poultry houses. (2) Dynamically adjust detection sensitivity based on real-time thermal readings. (3) Establish temperature-triggered alert thresholds to reduce false interventions. Implementation focus: Correlation between thermal stress and panting intensity.

Finally, to bridge the temporal modeling gap and enable behavior-level interpretation, we propose a phased approach specifically designed to integrate ethological benchmarks for distinguishing adaptive thermoregulation from heat-stress-induced dysfunction: (1) Short-term: Implement lightweight LSTM networks to analyze temporal sequences. Crucially, this will not only detect panting occurrence but also quantify its rhythm and duration. (2) Mid-term: Develop 3D-CNN architectures to process extended video clips for robust spatiotemporal feature fusion, facilitating continuous monitoring of panting bouts. (3) Long-term: Advance to transformer-based models that will explicitly correlate key behavioral metrics—particularly panting duration and its potential disruption of resting periods—with physiological stress biomarkers. This integration aims to establish quantitative thresholds: short-duration panting within the normal rhythm range signifies adaptive thermoregulation, while prolonged panting (exceeding physiologically adaptive timeframes) or panting coinciding with significant behavioral suppression (e.g., reduced resting) will be flagged as indicators of heat-stress-induced dysfunction and compromised welfare.

## 5. Conclusions

This study aimed to develop an efficient and lightweight object detection method (OM-YOLO) for accurately identifying open-mouth behavior in cage-raised broilers through image features—a critical visual indicator highly correlated with heat stress. The research objective was successfully achieved. By integrating a P2 detection layer, SGConv module, BIFPN structure, and SEAttention mechanism into the YOLOv8n architecture, OM-YOLO significantly enhanced its capability to detect broiler open-mouth actions while effectively preserving crucial spatial features. Evaluation results across multiple protocols confirmed the model’s strong generalization capability: on the independent test set, it achieved 0.920 mAP@50 with significant precision/recall improvements. After rigorous leakage-free retraining under strict data partitioning protocols, performance further increased to 0.927 mAP@50. Most importantly, this process provided definitive validation of OM-YOLO’s superiority, substantially and conclusively outperforming the baseline (0.879 mAP@50). Furthermore, OM-YOLO maintained its lightweight profile (only 2.53 million parameters and 11.6 GFLOPs) alongside these performance gains, demonstrating its potential for efficient deployment in practical farming environments. Ablation studies provided valuable supporting evidence for the effectiveness of each enhanced module and their synergistic effects, particularly under challenging conditions such as occlusion, high target density, and image noise. Consequently, OM-YOLO provided the poultry industry with a high-accuracy, low-computational-cost automated solution for detecting broiler open-mouth behavior, offering direct application value for computer vision-based real-time heat stress warning and environmental management.

## Figures and Tables

**Table 1:** The distribution of chicken mouth opening data during the experiment.

Data Source	The Number of Images	Train Set	Val Set	Test Set
Original	1000	400	300	300
Horizontal Flip	1000	1000	0	0
Random Cropping	1000	1000	0	0
Total	3000	2400	300	300

**Table 2:** Balanced distribution of growth stages across dataset partitions.

Growth Stage	Train Set	Val Set	Test Set
Brooding	771 (32%)	96 (32%)	98 (33%)
growth	814 (34%)	100 (33%)	102 (34%)
Fattening	816 (34%)	104 (35%)	100 (33%)
Total	2400	300	300

**Table 3:** Key software versions.

Software	Version
PyTorch	1.13.1
Ultralytics YOLOv8	8.3.55
CUDA Toolkit	11.7
OpenCV	4.10.0.84
Torchvision	0.14.1

**Table 4:** Method Hyperparameter Settings.

Hyperparameters	Value
Learning Rate	0.01
Image Size	640 × 640
Momentum	0.937
Optimizer	SGD
Batch Size	16
Epoch	200
Weight Decay	0.0005

**Table 5:** Detailed Results of Individual Module Experiments, Bold values indicate optimal results. (Results obtained before identifying data leakage issue, for reference only).

Models	P	R	mAP@50	mAP@50–95	Parameters (M)	GFlops
YOLOv8n	0.789	0.839	0.874	0.418	3.005	8.1
+BI	0.806	0.853	0.887	0.439	**1.991**	**7.1**
+ SGConv	**0.835**	0.791	0.882	0.433	2.639	7.3
+SEAttention	0.825	0.843	**0.894**	0.445	3.005	8.1
+P2	0.784	**0.855**	**0.894**	**0.446**	2.881	11.7

**Table 6:** Detailed Results of Combined Module Experiments, Bold values indicate optimal results. (Results obtained before identifying data leakage issue, for reference only).

Models	P	R	mAP@50	mAP@50–95	Parameters(M)	GFlops
YOLOv8n	0.789	0.839	0.874	0.418	3.005	**8.1**
+SGConv+P2	0.856	0.854	0.907	0.451	**2.51**	10.9
+SGConv+SE	0.836	0.828	0.901	0.436	2.64	9.7
+SGConv+BI	0.846	0.832	0.904	0.464	2.65	9.3
+P2+SE	0.833	0.856	0.906	0.458	2.88	11.7
+P2+BI	0.837	0.855	0.897	0.465	2.89	11.7
+BI+SE	0.837	0.827	0.899	0.43	3.02	**8.1**
+P2+BI+SE	0.824	0.871	0.903	0.463	2.90	11.7
+P2+BI+SGConv	0.803	**0.892**	0.913	0.459	2.53	11.9
+P2+SE+SGConv	0.81	0.882	0.915	0.471	2.52	10.9
+SE+BI+SGConv	0.844	0.864	0.912	0.470	2.66	10.3
+P2+BI+SE+SGConv (OM-YOLO)	**0.872**	0.873	**0.931**	**0.491**	2.53	11.6

**Table 7:** Detailed Experimental Results of Different Models, Bold values indicate optimal results. (Results obtained before identifying data leakage issue, for reference only).

Models	P	R	mAP@50	mAP@50–95	Parameters (M)	GFlops
Yolov5n	0.732	0.789	0.803	0.404	**2.503**	7.1
YOLOv8n	0.789	0.839	0.874	0.418	3.005	8.1
Yolov10n	0.751	0.764	0.813	0.374	2.69	8.2
Yolov11n	0.713	0.748	0.79	0.375	2.59	**6.4**
Faster-Rcnn	0.721	0.726	0.755	0.365	41.39	208
TOOD	0.784	0.786	0.814	0.379	32.04	199
OM-YOLO	**0.872**	**0.873**	**0.931**	**0.491**	2.53	11.6

**Table 8:** Performance Comparison Across Test Sets.

Test Set	P	R	mAP@50	F1-Score
Original Test Set *	0.872	0.873	0.931	0.872
Independent Test Set	0.868	0.860	0.920	0.864

* Note: The original test set suffers from data correlation issues (Section 2.1.3); results are for reference only.

**Table 10:** Performance comparison: Original vs. corrected partitioning.

Model	Partitioning	mAP@50	ΔmAP@50
YOLOv8n	Original	0.874	
	Corrected	0.879	+0.5%
OM-YOLO	Original	0.931	
	Corrected	0.927	−0.4%

## Data Availability

The data provided in this study are available upon request from the corresponding author.

## Supplementary Materials

The following supporting information can be downloaded at: https://www.mdpi.com/article/10.3390/ani15182732/s1, Table S1: Augmentation metadata; Table S2: Complete data partition; Supplementary Note S1: Step-by-step reproducibility guide.
