# Supplementary material for: Development of a Tool to Detect Open-Mouthed Respiration in Caged Broilers
Source: Animals (Basel). 2025 Sep 18;15(18):2732. doi: 10.3390/ani15182732 (PMC12466475; doi:10.3390/ani15182732)
Supplement: Supplementary file 1 [file animals-15-02732-s001.zip › animals-3747024-supplementary/Supplementary/Supplementary Note S1.pdf]

Reproducibility Documentation

Random Seed Implementation

All random operations used fixed seeds:

- Data splitting: `seed=42`
- Data augmentation: `seed=42`

Data Splitting Code

```
import numpy as np
from sklearn.model_selection import train_test_split

# ===== FIXED SEEDS ===== #
np.random.seed(42)  # Global seed

# Load all 1000 original images
originals = [f"IMG_{i:03d}.jpg" for i in range(1, 1001)]

# Step 1: Split original images (8:1:1)
train_val, test = train_test_split(originals, test_size=300, random_state=42)
train, val = train_test_split(train_val, test_size=300, random_state=42)

# Step 2: Generate augmentations ONLY for training set
train_aug_flip = [f"{img}_flip.jpg" for img in train]  # Horizontal flip
train_aug_crop = [f"{img}_crop.jpg" for img in train]  # Random crop

# Final sets
train_set = train + train_aug_flip + train_aug_crop
val_set = val
test_set = test
```
