# Supplementary figures and images for: Development of a Tool to Detect Open-Mouthed Respiration in Caged Broilers
Source: Animals (Basel). 2025 Sep 18;15(18):2732. doi: 10.3390/ani15182732 (PMC12466475; doi:10.3390/ani15182732)

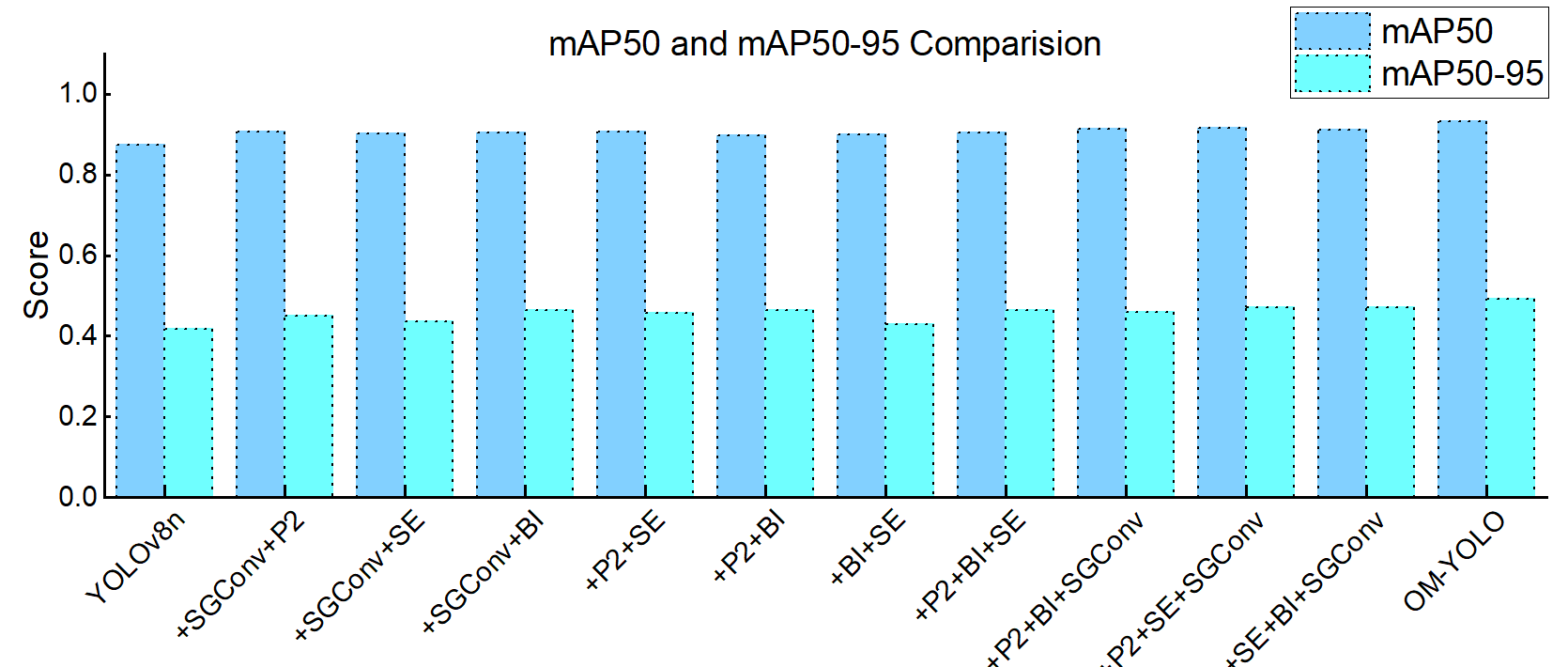

Supplement: Supplementary file 1 [file animals-15-02732-s001.zip › animals-3747024-supplementary/Supplementary/Orginal figures/Figure 10.png]

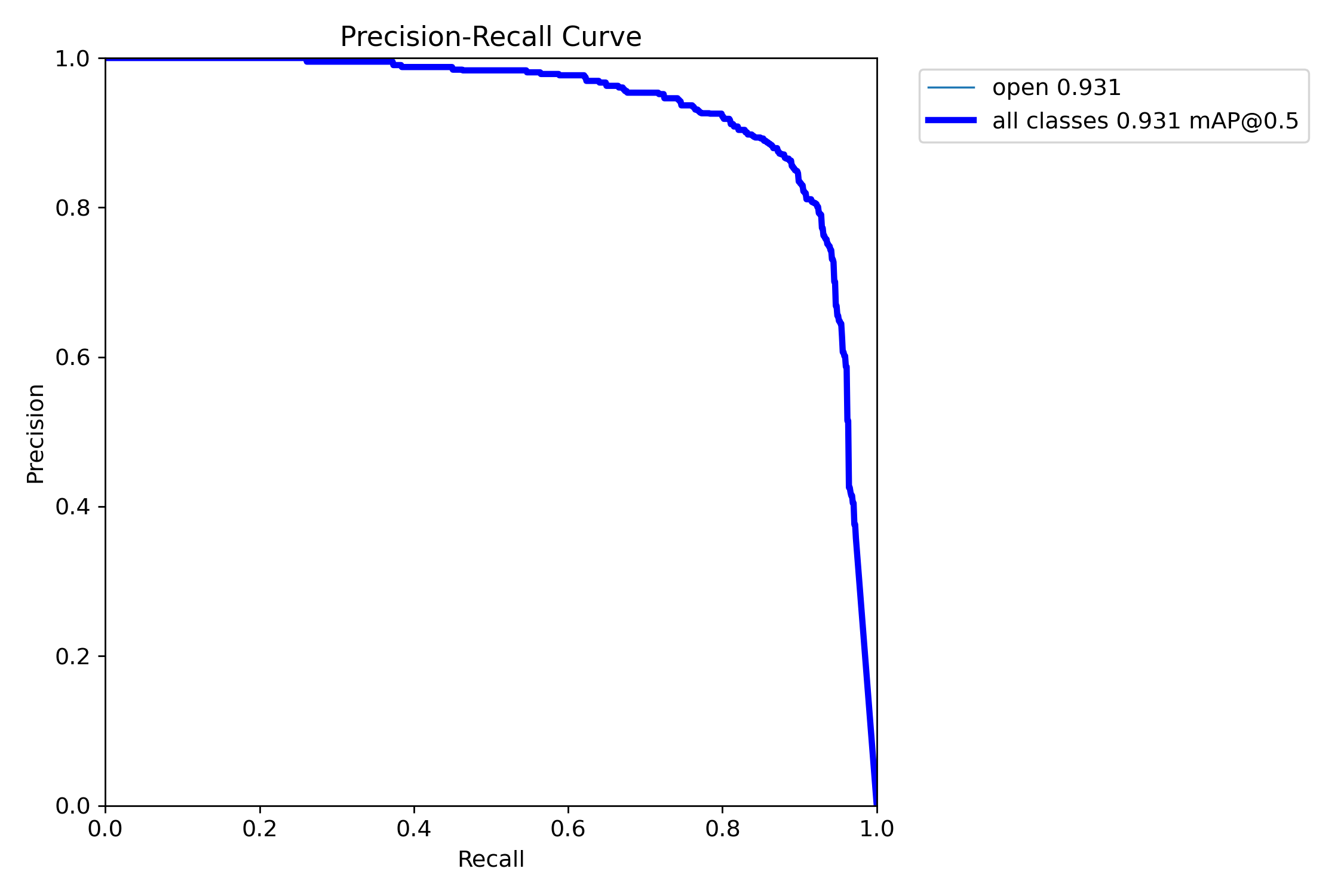

Supplement: Supplementary file 1 [file animals-15-02732-s001.zip › animals-3747024-supplementary/Supplementary/Orginal figures/Figure 11(b).png]

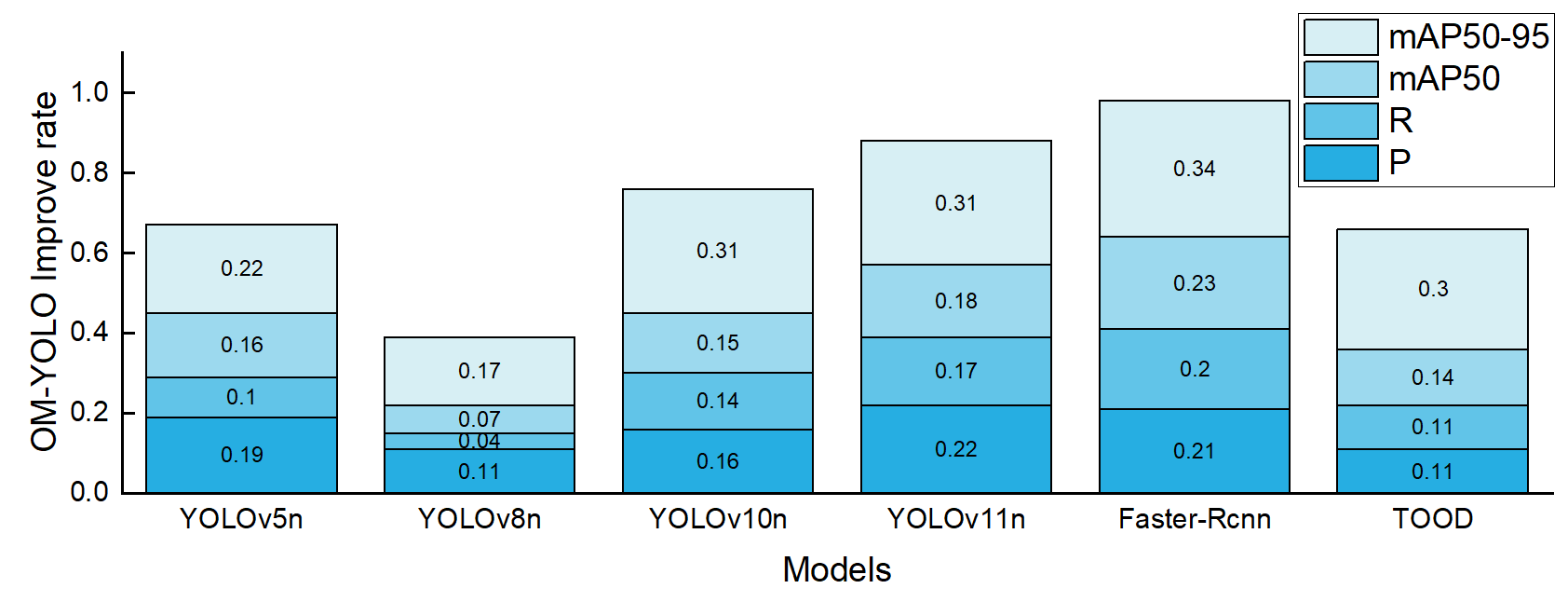

Supplement: Supplementary file 1 [file animals-15-02732-s001.zip › animals-3747024-supplementary/Supplementary/Orginal figures/Figure 12.png]

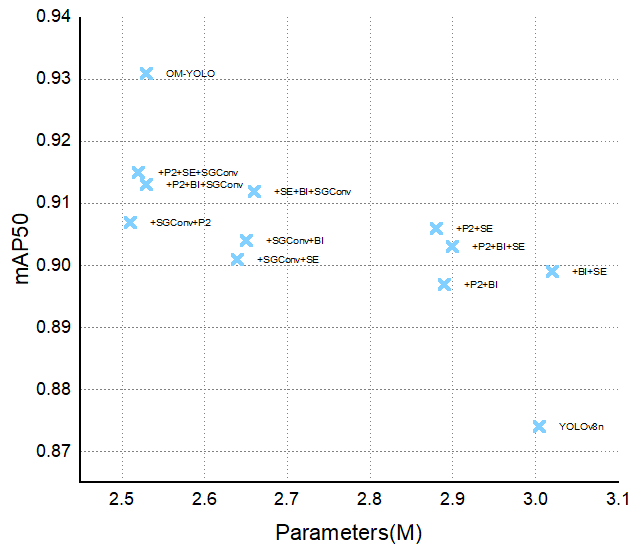

Supplement: Supplementary file 1 [file animals-15-02732-s001.zip › animals-3747024-supplementary/Supplementary/Orginal figures/Figure 15(a).png]

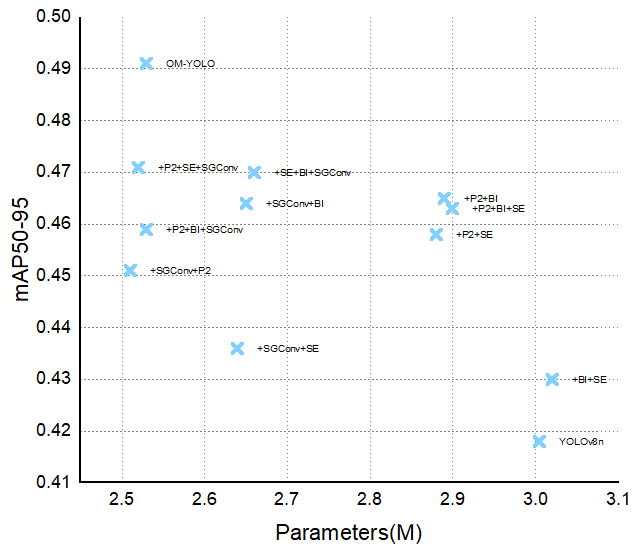

Supplement: Supplementary file 1 [file animals-15-02732-s001.zip › animals-3747024-supplementary/Supplementary/Orginal figures/Figure 15(b).png]

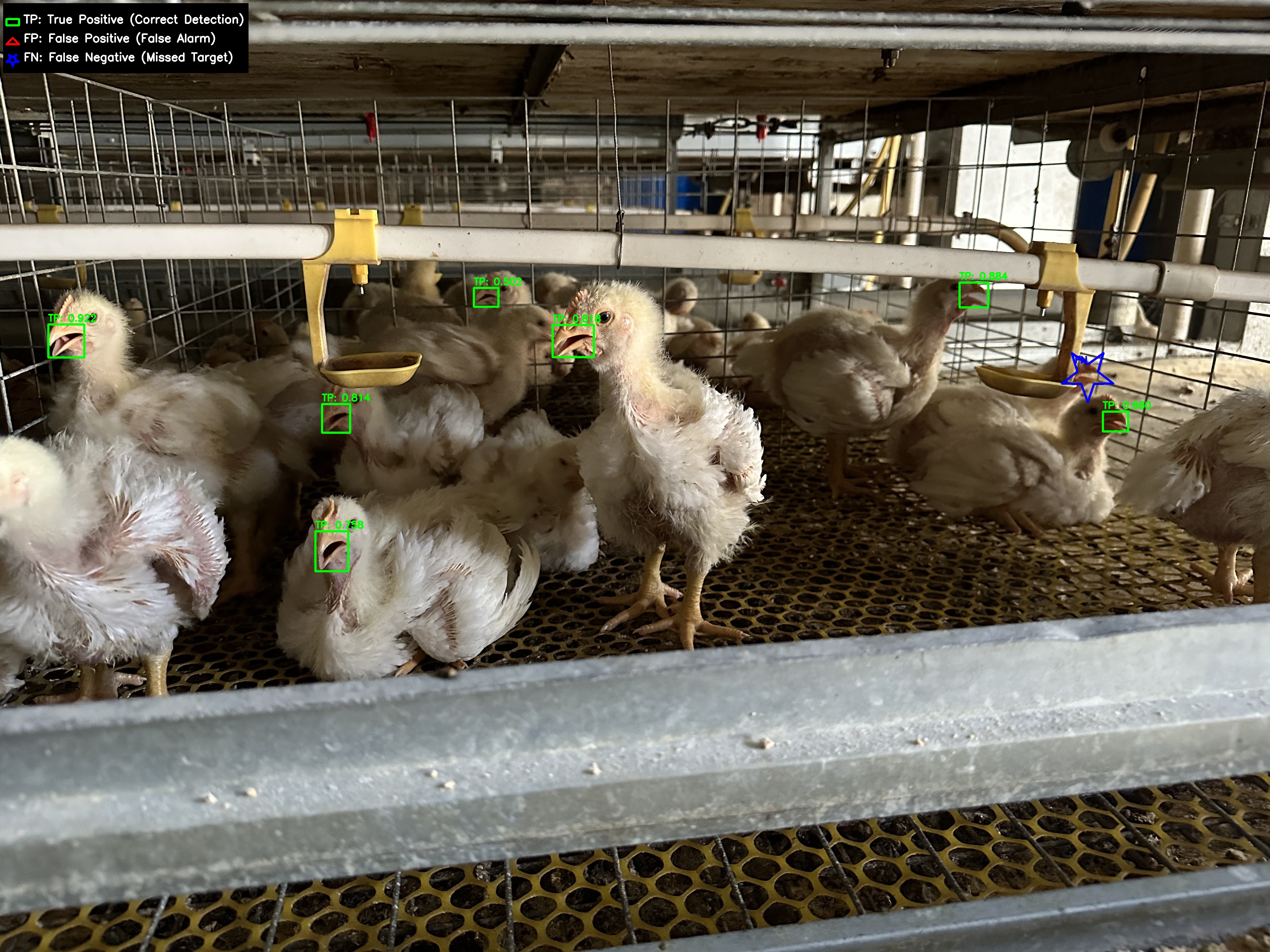

Supplement: Supplementary file 1 [file animals-15-02732-s001.zip › animals-3747024-supplementary/Supplementary/Orginal figures/Figure 16(a).JPG]

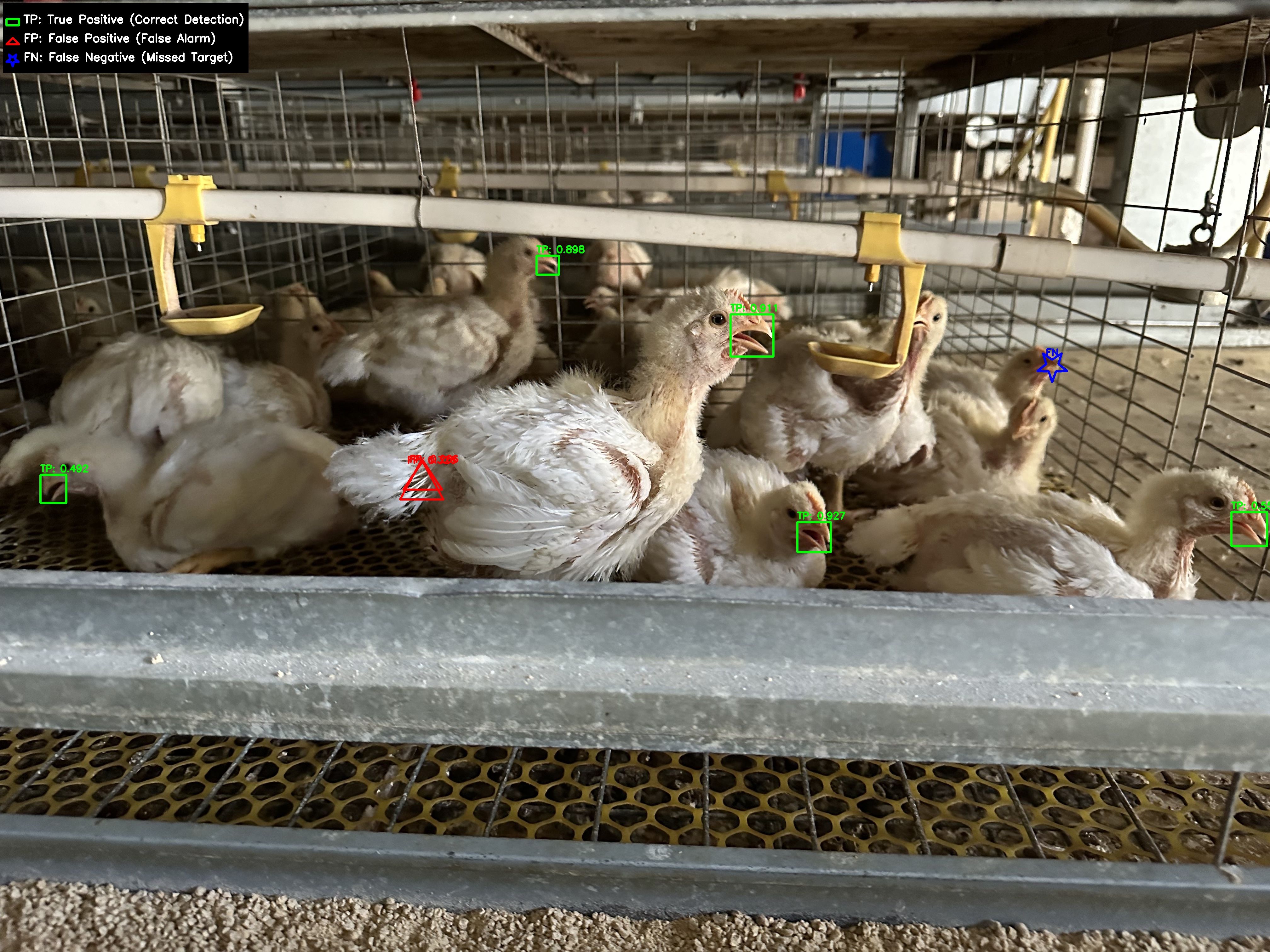

Supplement: Supplementary file 1 [file animals-15-02732-s001.zip › animals-3747024-supplementary/Supplementary/Orginal figures/Figure 16(b).JPG]

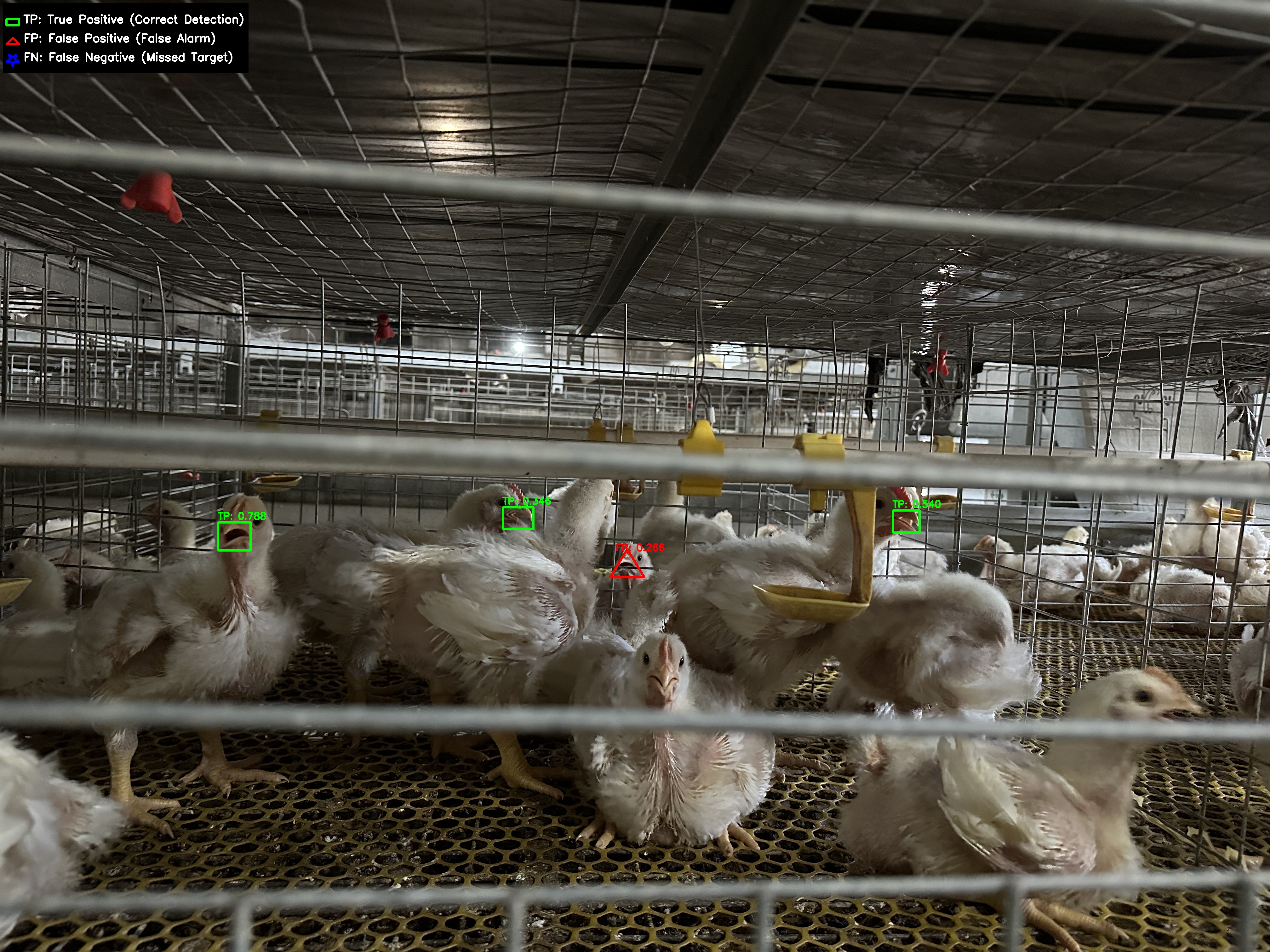

Supplement: Supplementary file 1 [file animals-15-02732-s001.zip › animals-3747024-supplementary/Supplementary/Orginal figures/Figure 16(c).JPG]

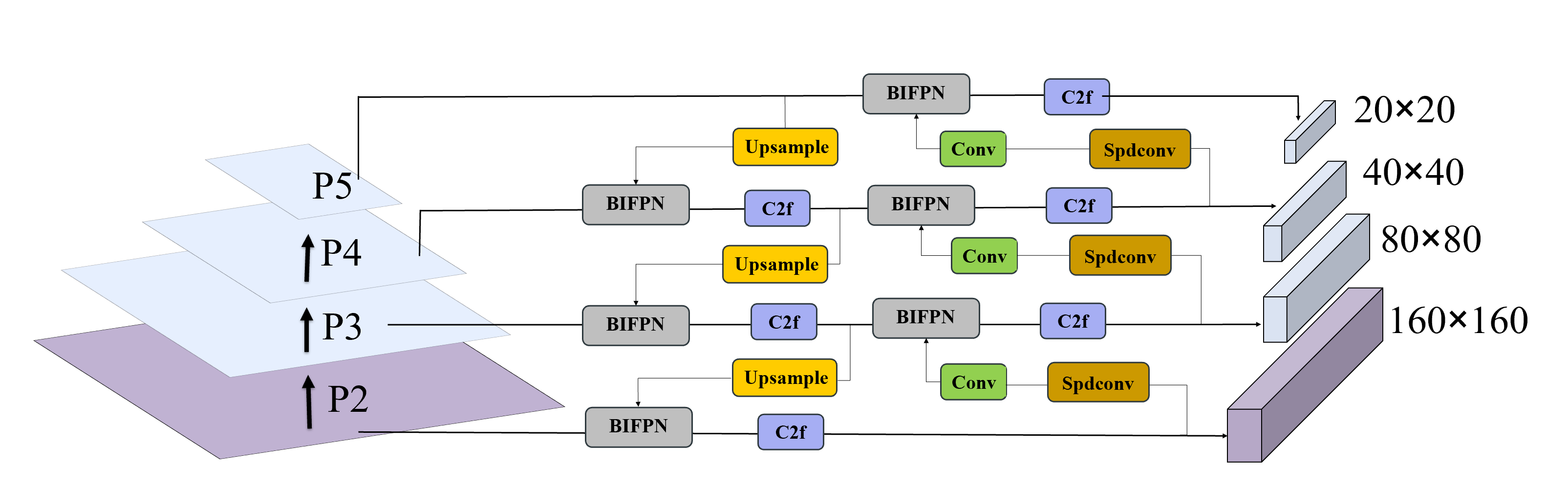

Supplement: Supplementary file 1 [file animals-15-02732-s001.zip › animals-3747024-supplementary/Supplementary/Orginal figures/Figure 3.png]

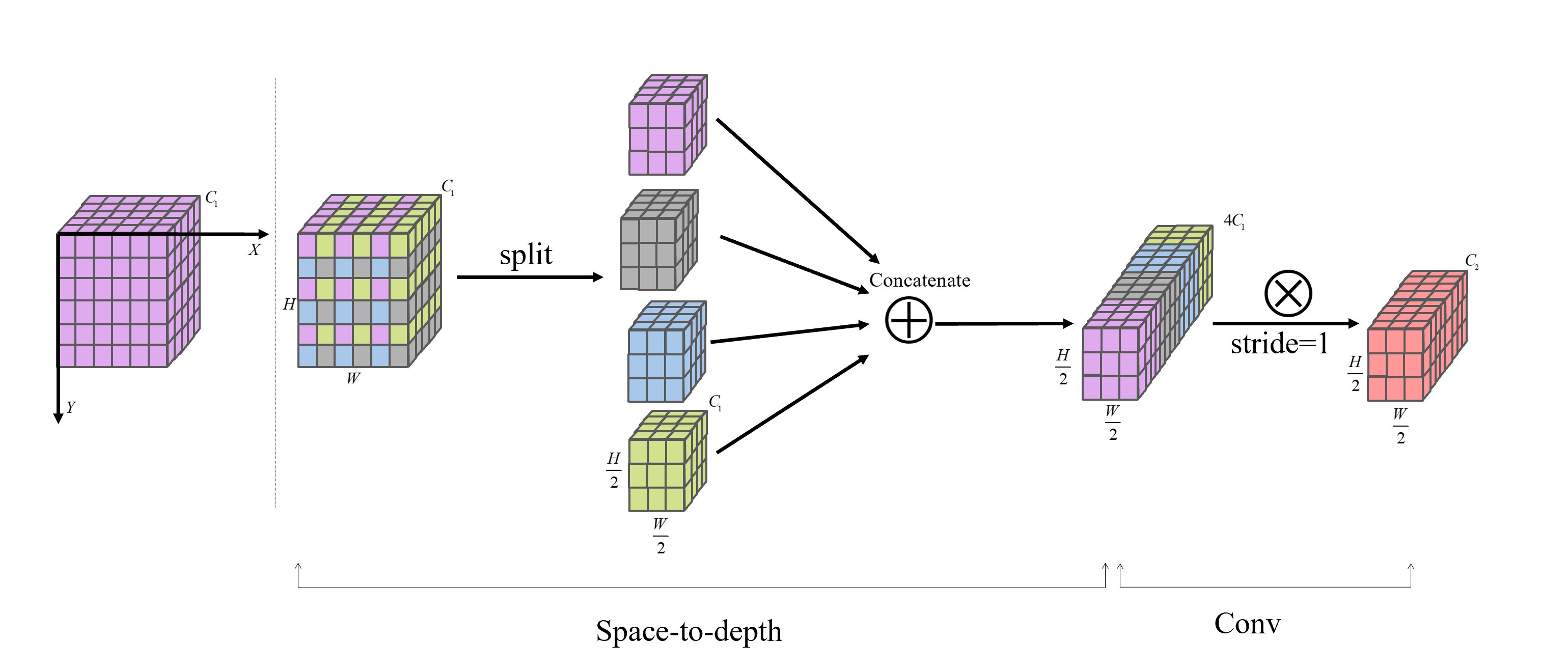

Supplement: Supplementary file 1 [file animals-15-02732-s001.zip › animals-3747024-supplementary/Supplementary/Orginal figures/Figure 4(a).png]

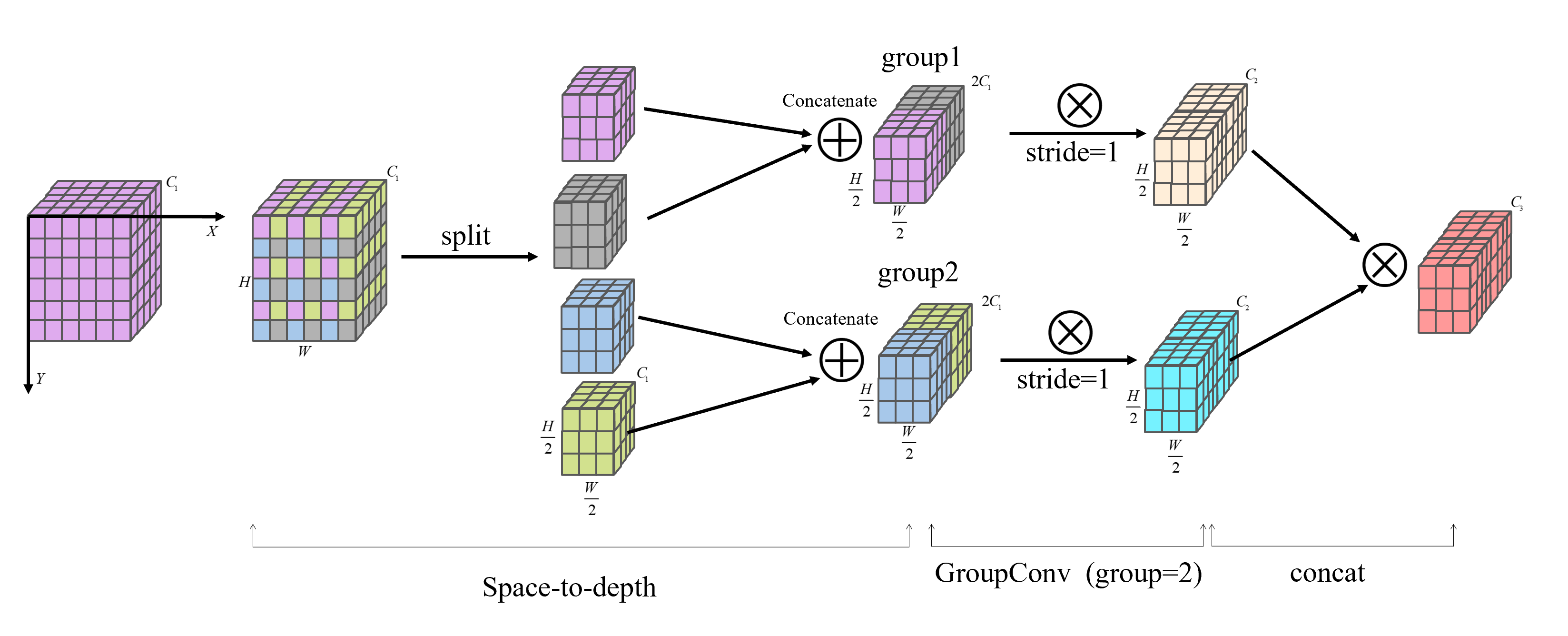

Supplement: Supplementary file 1 [file animals-15-02732-s001.zip › animals-3747024-supplementary/Supplementary/Orginal figures/Figure 4(b).png]

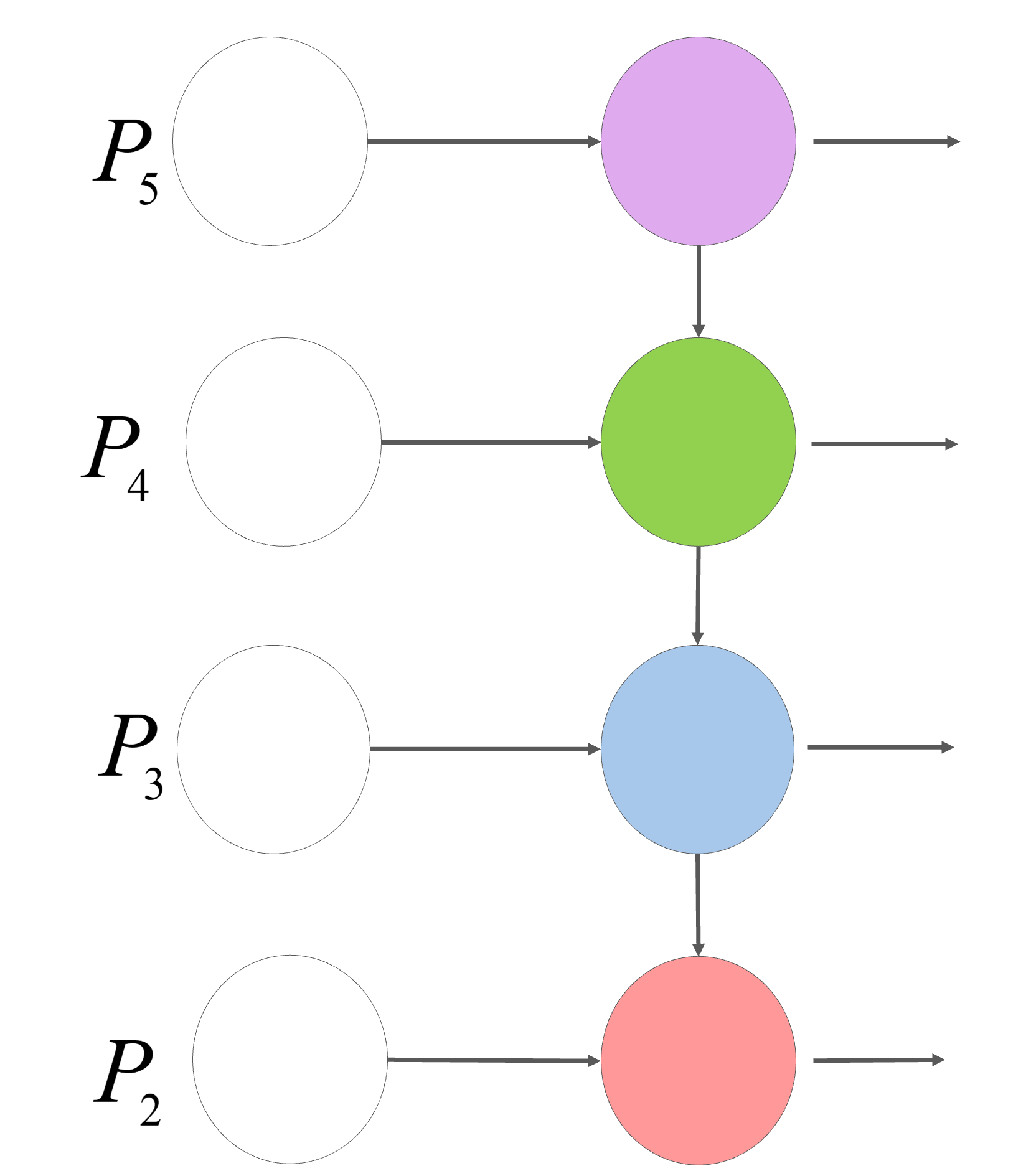

Supplement: Supplementary file 1 [file animals-15-02732-s001.zip › animals-3747024-supplementary/Supplementary/Orginal figures/Figure 5(a).png]

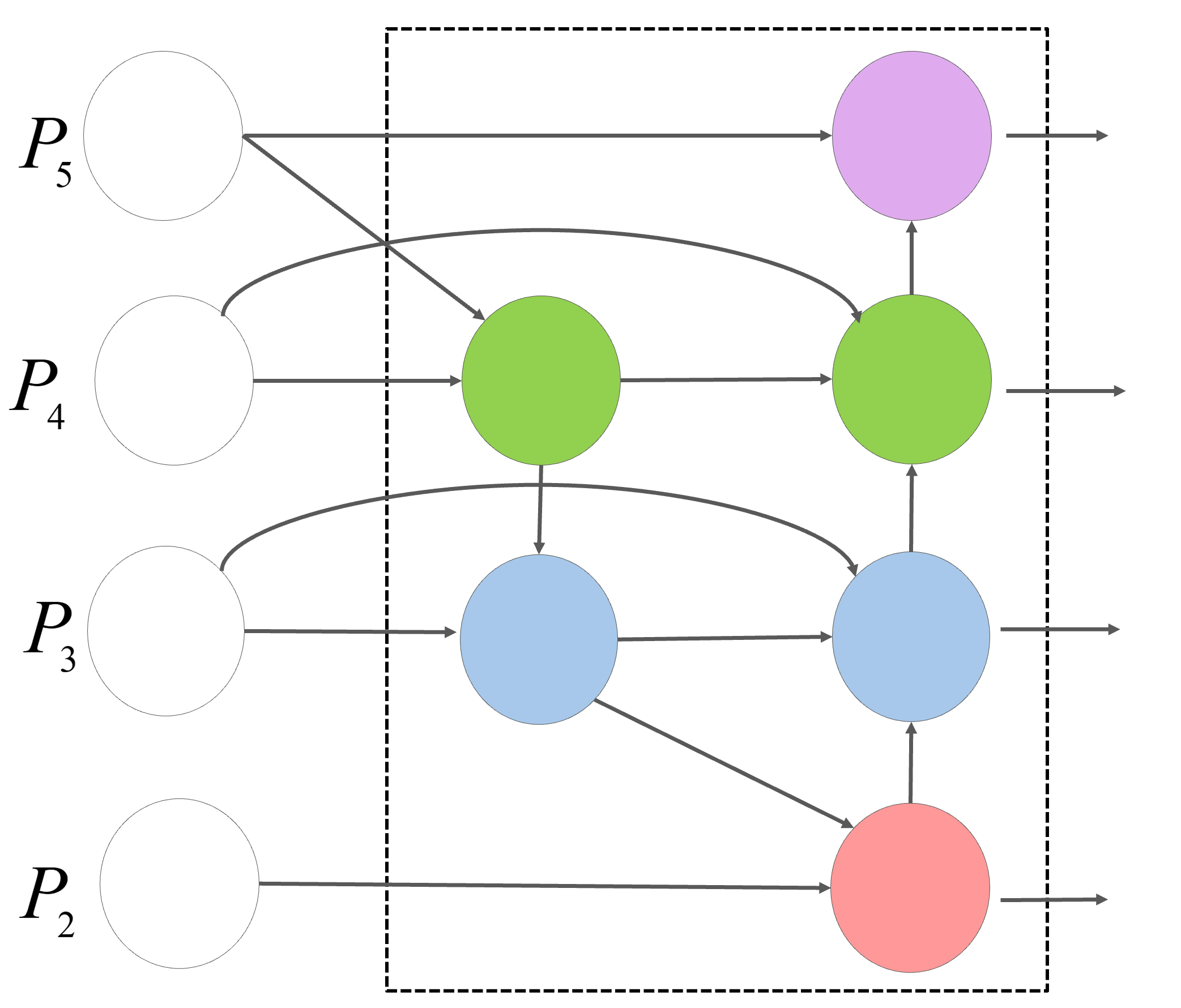

Supplement: Supplementary file 1 [file animals-15-02732-s001.zip › animals-3747024-supplementary/Supplementary/Orginal figures/Figure 5(b).png]

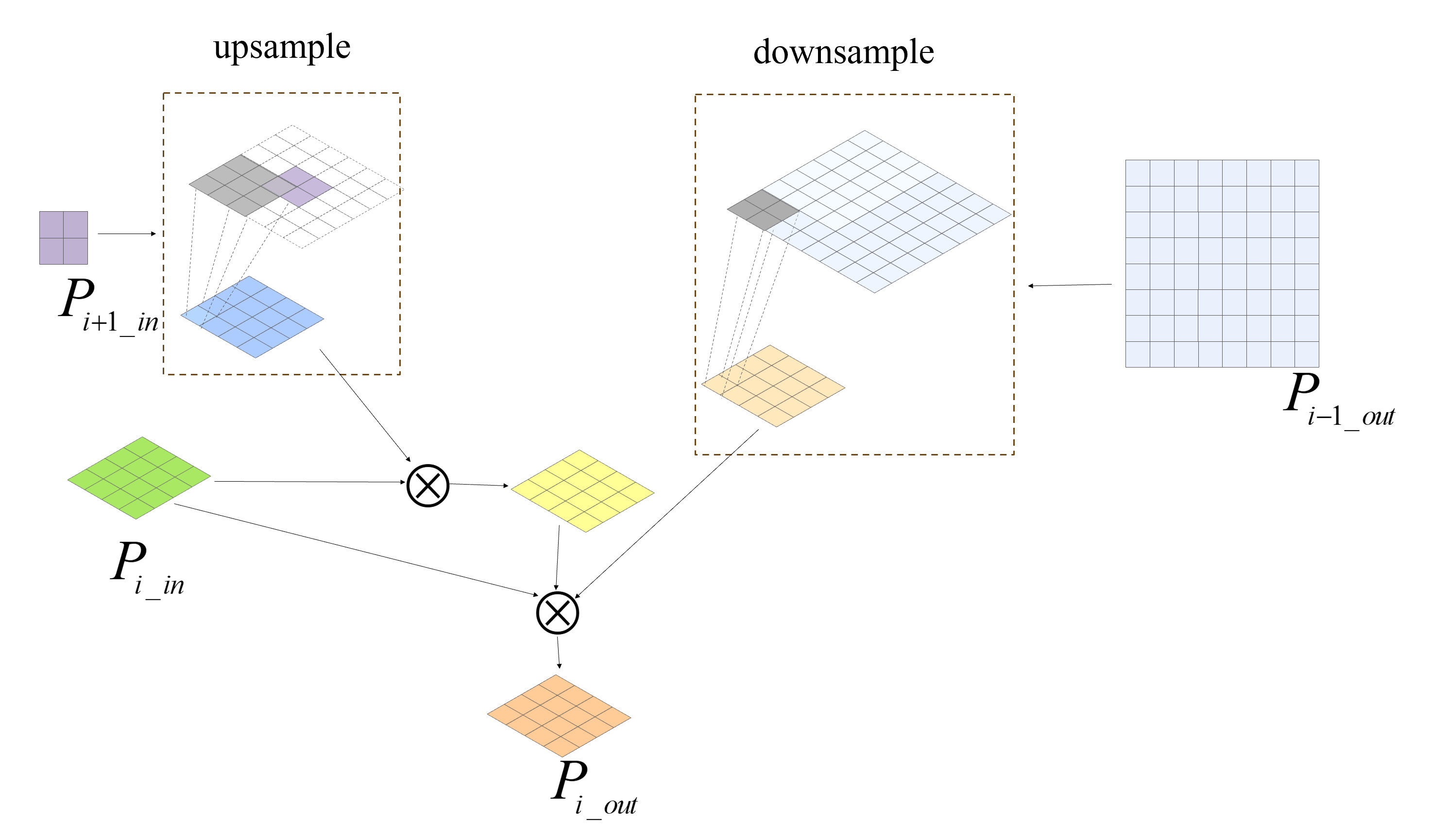

Supplement: Supplementary file 1 [file animals-15-02732-s001.zip › animals-3747024-supplementary/Supplementary/Orginal figures/Figure 5(c).png]

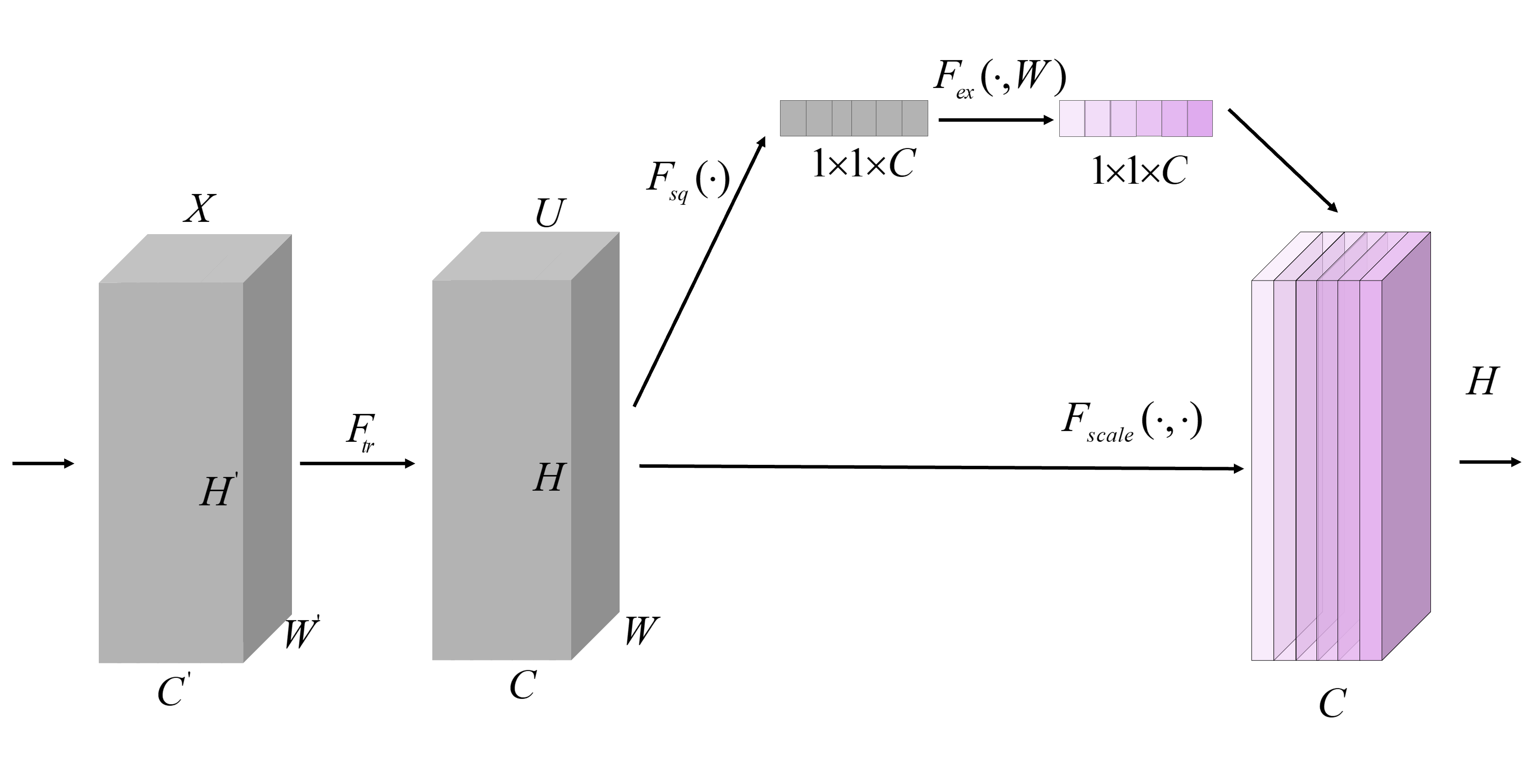

Supplement: Supplementary file 1 [file animals-15-02732-s001.zip › animals-3747024-supplementary/Supplementary/Orginal figures/Figure 6(a).png]

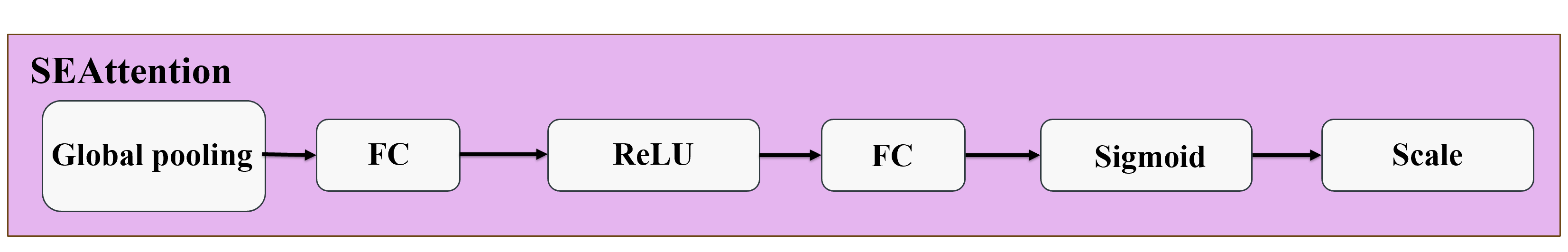

Supplement: Supplementary file 1 [file animals-15-02732-s001.zip › animals-3747024-supplementary/Supplementary/Orginal figures/Figure 6(b).png]

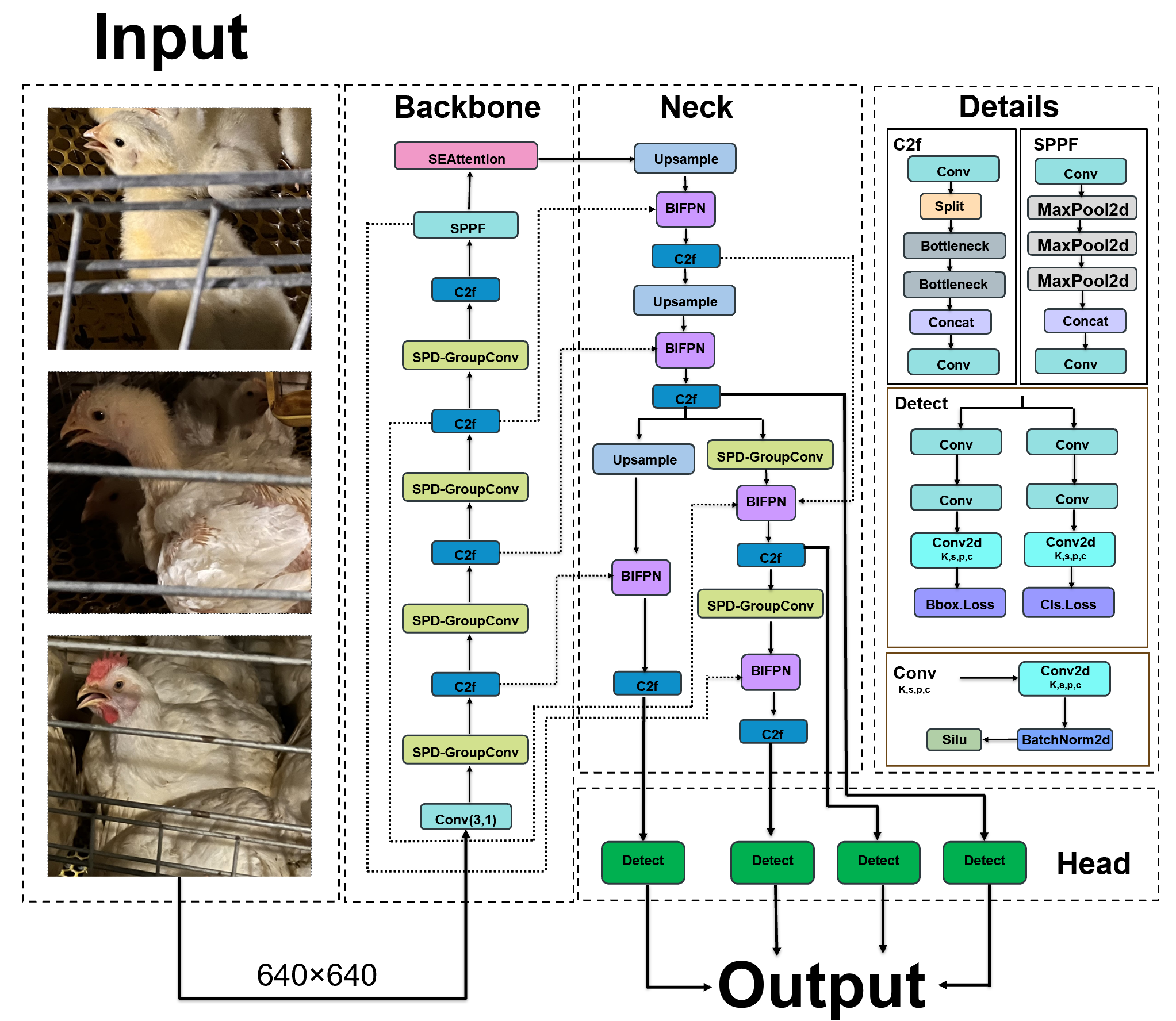

Supplement: Supplementary file 1 [file animals-15-02732-s001.zip › animals-3747024-supplementary/Supplementary/Orginal figures/figure 7.png]

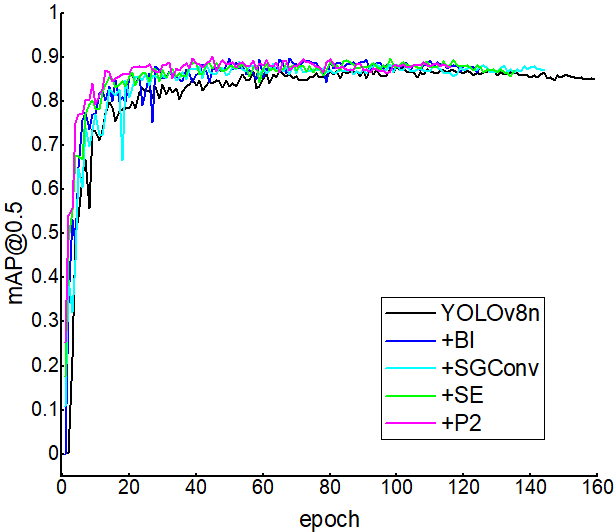

Supplement: Supplementary file 1 [file animals-15-02732-s001.zip › animals-3747024-supplementary/Supplementary/Orginal figures/Figure 8(a).png]

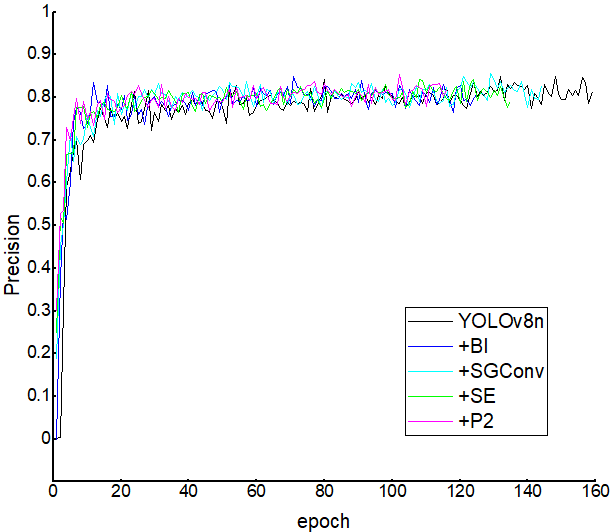

Supplement: Supplementary file 1 [file animals-15-02732-s001.zip › animals-3747024-supplementary/Supplementary/Orginal figures/Figure 8(b).png]

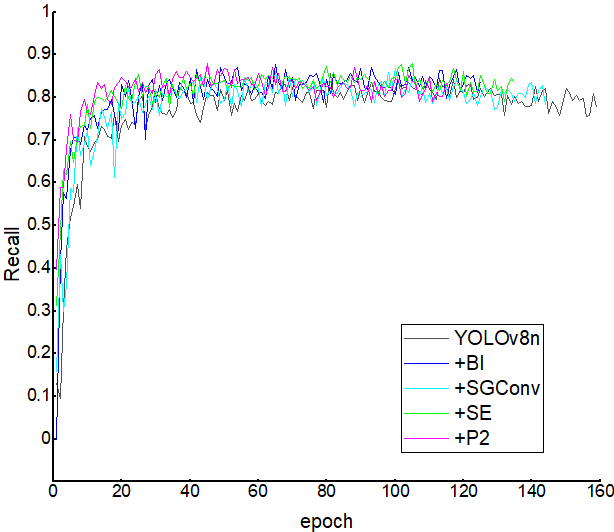

Supplement: Supplementary file 1 [file animals-15-02732-s001.zip › animals-3747024-supplementary/Supplementary/Orginal figures/Figure 8(c).png]
